# Transcutaneous auricular vagus nerve stimulation alleviates anxiety-like behaviors in mice with post-traumatic stress disorder by regulating glutamatergic neurons in the anterior cingulate cortex

**DOI:** 10.1038/s41398-025-03535-9

**Published:** 2025-08-23

**Authors:** Zhijun Diao, Yan Zuo, Jinming Zhang, Ke Chen, Yongbin Liu, Yuwei Wu, Feng Miao, Haifa Qiao

**Affiliations:** 1 Institute for Chinese Medicine Frontier Interdisciplinary Science and Technology, Shaanxi University of Chinese Medicine, Xianyang, Shaanxi Province 712046 China; 2 Shaanxi Key Laboratory of Integrative Acupuncture & Medicine, Shaanxi University of Chinese Medicine, Xianyang, Shaanxi Province 712046 China; 3 Key Laboratory of Acupuncture and Neurobiology, Shaanxi Administration of Traditional Chinese Medicine, Xianyang, Shaanxi Province 712046 China; 4 College of Acupuncture-moxibustion and Tuina, Shaanxi University of Chinese Medicine, Xianyang, Shaanxi Province 712046 China; 5 Key Laboratory of Modern Teaching Technology, Ministry of Education, Shaanxi Normal University, Xi’an, Shaanxi Province 710062 China; 6 The Second Affiliated Hospital of Shaanxi University of Chinese Medicine, Xianyang, Shaanxi Province 712020 China

**Keywords:** Neuroscience, Psychiatric disorders

## Abstract

Vagus nerve stimulation has been certified to be an effective therapeutic modality for emotional disorders, especially anxiety triggered by post-traumatic stress disorder (PTSD). Nevertheless, the neural mechanisms underlying the efficacy of transcutaneous auricular vagus nerve stimulation (taVNS) remain poorly understood. In this study, we aimed to elucidate whether and how taVNS influences anxiety-like behaviors elicited by PTSD, focusing on synaptic plasticity in taVNS-activated neurons (TANs) of the anterior cingulate cortex (ACC). Our findings substantiate that taVNS significantly mitigates anxiety-like behaviors in PTSD-like male mice via activating specific glutamatergic neurons in the ACC. Notably, these glutamatergic TANs^ACC^ exhibited marked enhancements in presynaptic excitatory transmission relative to those non-activated glutamatergic neurons in the ACC. This enhancement of presynaptic release further prevented the induction of presynaptic long-term potentiation (pre-LTP), manifesting as presynaptic depotentiation. Furthermore, inhibiting these glutamatergic TANs^ACC^ weakened the positive effects of taVNS on anxiety-like behaviors in PTSD-like male mice. Conversely, activating these glutamatergic TANs^ACC^ did not further amplify the effects of taVNS on anxiety-like behaviors. Collectively, our results reveal that the upregulation of presynaptic transmission in glutamatergic TANs^ACC^ is responsible for the positive effects of taVNS on anxiety-like behaviors in PTSD-like male mice, providing new insights into functional and activity patterns of the specific brain regions involved in the effects of taVNS.

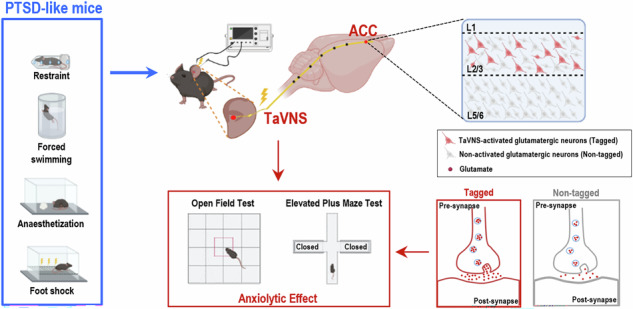

## Introduction

Posttraumatic stress disorder (PTSD) is a debilitating neuropsychiatric disorder triggered by extreme accidents or disasters [1], which generally leads to concomitant affective disorders, such as anxiety [2]. PTSD frequently elicits anxiety, which in turn further increases its severity [3]. The cycle of worsening PTSD and anxiety is difficult to treat in a clinical setting. Therefore, improvement of anxiety state in PTSD patients is a key factor in shortening the course of the disease. Statistically, the lifetime occurrence rate of PTSD worldwide varies between 1.9% and 6.8% [1], comorbidity rates for anxiety range from 39% to 97% [3], and as many as 40% of the patients may not respond to pharmacological or traditional exposure therapies [1]. Thus, it is crucial to understand the underlying pathological mechanisms responsible for the dysregulated anxiety in PTSD to develop more effective clinical treatments.

Transcutaneous auricular vagus nerve stimulation (taVNS) has been recognized as a promising intervention for various neuropsychiatric disorders, such as epilepsy, migraine, and depression [4]. According to anatomic research, the only vagus nerve (VN) segment located on the body’s surface is the auricular branch of the VN [4]. This branch is primarily distributed in the outer ear canal and concha, which includes the cymba conchae and cavum concha [5]. It has been reported that VN stimulation (VNS) reduced PTSD-like symptoms in rats and improved scores on the Hamilton Anxiety Scale in human patients with depression [6, 7]. However, it is still unclear whether stimulating the auricular vagus nerve (AVN) can alleviate PTSD-related symptoms, particularly anxiety.

It is assumed that stimulation of the AVN activates the nucleus tractus solitarius (NTS), which subsequently affects cortical and subcortical structures, for instance, the parabrachial nucleus, hippocampus, thalamus, prefrontal cortex (PFC), amygdala, and anterior cingulate cortex (ACC) [8]. These regions are important nodes of the emotional regulation network. Thus, the VN directly and indirectly interacts with the emotion-associated neural circuits, influencing their activities. ACC is a pivotal region for integrating sensory perception and emotional responses [9]. The structural and functional damage to the ACC may lead to heightened fear and other adverse emotions [10]. At the same time, VNS significantly relieves PTSD-related symptoms by activating the ACC in both individuals and animals [11–13]. Although the ACC has been identified as the core brain structure in both pathological anxiety of PTSD and the effects of VNS, the intricate mechanisms underlying its roles have remained unknown. For instance, it is still unclear whether and how taVNS can correct PTSD-related anxiety behaviors by modulating ACC activity, and which type of ACC neuronal ensembles can be recruited through taVNS.

To study this, we induced a modified single prolonged stress (mSPS) in mice to simulate exposure to PTSD-like stresses, followed by taVNS treatments to observe the effects on anxiety-like behaviors. Hence, we hypothesized that taVNS could reduce anxiety in mice with mSPS, with the underlying mechanism potentially involving functional changes within a specific neuronal ensemble in the ACC. To evaluate our hypothesis, we applied multifaceted approaches encompassing behavioral assessments, the Fos-targeted recombination in active populations (Fos-TRAP) labeling strategy, electrophysiology, and chemogenetic manipulation methodologies. These findings will not only shed light on the underlying mechanisms of taVNS in neuropsychiatric disorders but also provide new insights into functional connectivity and activity patterns of the specific brain regions involved in the effects of taVNS.

## Materials and methods

### Animals

Adult male C57BL/6 J mice were procured from Beijing Vital River Laboratory Animal Technology Co., Ltd. The ROSA26 Cre-dependent tdTomato (Rosa26-LSL-tdTomato, Ai14) reporter mice and Fos-TRAP2 (Fos^2A-iCreER^) mice were generously provided by Prof. Jing Han (Shaanxi Normal University). Additionally, Fos-TRAP2 mice were interbred with Rosa26-tdTomato mice to yield transgenic mice exhibiting cell-type-specific red tdTomato-expressing (Fos-tdTomato) neurons, referred to as RosaFos2 mice. All randomly selected male mice aged two to three months were used for these studies. Four to five mice were housed per cage and subjected to a 12-h light/dark cycle (lights on at 8:00 a.m.) in a temperature-controlled environment with unrestricted access to food and water. The described experimental procedures received approval from the Animal Experiment Ethics Committee of Shaanxi University of Chinese Medicine (China) (No. SUCMDL20220304003). All experiment protocols were conducted following the National Institutes of Health (NIH) Guide for the Care and Use of Laboratory Animals.

### Animal model induced by mSPS

The PTSD-like mouse model was established via an mSPS protocol incorporating four consecutive stressors: restraint stress, forced swimming, deep anesthesia, and unconditioned foot shock, as described in the previous report [1] with minor modifications and detailed in the Supplemental Material.

### Stimulation protocol of taVNS

TaVNS commenced after a week of mSPS exposure with mice, as previously described [14–16]. The taVNS and mSPS+taVNS groups received taVNS stimulation, while the Sham and mSPS+Sham groups underwent sham stimulation. Mice were anesthetized with 2% isoflurane and maintained at 1% to minimize procedural stress. Positive and negative electrodes (0.16 × 7 mm sterile stainless-steel needles), equipped with the HANS Acupoint Nerve Stimulator (HANS-200A, Nanjing Jisheng Medical Technology Co., Ltd., Nanjing, China), were inserted approximately 1–2 mm deep into the auricular nail cavities of the taVNS and mSPS+taVNS groups. Successful delivery of the stimulus was indicated by slight fluttering in the external auricle. Both groups of mice received continuous-mode stimulation for 30 min daily over 6 consecutive days, delivering a 1 mA current with a frequency of 2/15 Hz (2 and 15 Hz, switched every second). For the Sham and mSPS+Sham group, sham stimulation was performed by placing needles on the helix of the auricle outside of the AVN innervated area, without applying any electrical current, which served as the negative control. All other conditions were identical to those of the taVNS and mSPS+taVNS groups. Throughout the taVNS period, the changes in body weight of the mice were measured, along with monitoring their body temperature and feed intake every two days.

### Activity-dependent neural tagging

Population tagging was conducted via intraperitoneal (i.p.) injection of 4-hydroxytamoxifen (4-OHT) immediately following sham stimulation or taVNS. The 4-OHT (10 mg/ml, Cat#H6278, Sigma) was prepared as previously described [17] and detailed in the Supplemental Material. Mice received an i.p. injection of 50 mg/kg. The vehicle comprised identical components to the 4-OHT solution and was administered in the same volume. Following neural tagging, the mice were left undisturbed in their home cages to minimize the potential for non-specific recombination.

### Viruses and stereotactic microinjections

The viral vectors utilized in this study, including AAV-CMV-DIO-taCasp3-TEVp, AAV-EFlα-DIO-mCherry, AAV-CaMKIIα-EGFP, AAV-CaMKIIα-DIO-hM4Di-mCherry, and AAV-CaMKIIα-DIO-hM3Dq-mCherry, were obtained from BrainVTA, China, and administered bilaterally into the ACC at coordinates: A/P, +1.40 mm from bregma, M/L, ±0.20 mm, D/V, −2.00 mm. The AAV-CaMKIIα-DIO-mCherry or AAV-CMV-DIO-EGFP virus was employed as the control. All vectors were of the AAV2/9 serotype with a viral titer of (2–7) × 10^12^ particles per ml. Behavioral assays and electrophysiological recordings were conducted at least two weeks after viral injection. For chemogenetic modulation utilizing the designer receptors exclusively activated by designer drugs (DREADD) approach, clozapine-N-oxide (CNO, BrainVTA) was dissolved in 0.9% saline and administered (i.p.) at a dosage of 5 mg/kg, 40 min before behavioral assessments. If the virus injection was inaccurate, the animals or data points were systematically excluded from the analyses.

### Evaluation of anxiety-like behaviors

Assessments of anxiety-like behavioral tests were performed as described in our prior study [18, 19]. All behavioral tests were performed with investigators blinded to group assignments throughout data collection and analyses to minimize assessment bias. Initially, mice were acclimated to the experimental environment for 1 h. Subsequently, the movement trajectories of the mice were captured using a video tracking system (EthoVision XT, Noldus, Netherlands) and analyzed offline. In the open field test (OFT), the number of entries, duration within, and the distance traveled in the central zone were recorded for 10 min and calculated offline. In the marble burying test (MBT), twelve marbles were evenly arranged in a 3 × 4 grid within a plastic cage filled with 5 cm of sawdust. The number of marbles buried, defined as having over 75% of their surface covered by bedding material, was counted after each 20-min session [18, 19]. For the light and dark box test (LDBT), mice were placed in the center of the light compartment (occupying 2/3 of the 46 × 28 × 28 cm apparatus) and allowed to freely explore both chambers connected by a tunnel for 5 min. Time spent in the light compartment was recorded. In the elevated plus maze test (EPMT), the number of entries and the time spent within open arms were documented for 5 min and calculated offline [18], with the anxiety index analyzed as described below [20]. In the three-chamber test (TCT), social interaction capabilities were gauged [19, 21], and sniffing time was calculated offline. Additionally, the social index, denoting the differential engagement with the stranger versus an empty chamber relative to total engagement time, was calculated as previously described [22].

$${\rm{Anxiety}}\,{\rm{index}}=1-\frac{\frac{{\rm{Open}}\,{\rm{arm}}\,{\rm{time}}}{{\rm{Test}}\,{\rm{duration}}}\,+\,\frac{{\rm{Open}}\,{\rm{arm}}\,{\rm{entries}}}{{\rm{Total}}\,{\rm{number}}\,{\rm{of}}\,{\rm{entries}}}}{2}$$

$${\rm{Social}}\,{\rm{index}}=\frac{{\rm{Time}}\,{\rm{in}}\,{\rm{the}}\,{\rm{stranger}}\,{\rm{chamber}}-{\rm{Time}}\,{\rm{in}}\,{\rm{the}}\,{\rm{empty}}\,{\rm{chamber}}\,}{{\rm{Time}}\,{\rm{in}}\,{\rm{the}}\,{\rm{stranger}}\,{\rm{chamber}}+{\rm{Time}}\,{\rm{in}}\,{\rm{the}}\,{\rm{empty}}\,{\rm{chamber}}}$$

### Immunofluorescence

Mice were sacrificed and processed following established protocols [23] and detailed in the Supplemental Material. Their brain slices were prepared for free-floating fluorescence immunohistochemistry staining using primary and secondary antibodies. Fluorescence signals were subsequently detected and analyzed.

### Electrophysiological recording

Coronal brain slices encompassing the ACC region for whole-cell patch-clamp recordings were meticulously prepared following our previously established methodologies [9, 18, 19] and detailed in Supplemental Material. The glutamatergic taVNS-activated neurons (tagged neurons) were targeted based on EGFP and tdTomato fluorescence, while non-activated glutamatergic neurons (non-tagged neurons) of ACC were targeted only by EGFP fluorescence. The miniature excitatory postsynaptic current (mEPSC), evoked excitatory postsynaptic current (eEPSC), paired-pulse ratio (PPR), AMPAR/NMDAR ratio, pre-LTP, and post-LTP were recorded and analyzed by pClamp 10.5 software (Molecular Devices, USA).

For miniature excitatory postsynaptic current (mEPSC) recording, cellular membrane potentials were maintained at −60 mV in the presence of 1 µM tetrodotoxin (TTX, Chengdu Must Bio-Technology Co., LTD., China). After establishing a stable recording, the ten-minute-long mEPSC data were acquired in the gap-free mode. For evoked excitatory postsynaptic current (eEPSC) recordings, eEPSC from layer II/III neurons were elicited using a stainless-steel bipolar stimulating electrode positioned in layer V/VI of the ACC, with amplitudes adjusted between 50-100 pA to stabilize baseline recordings. The paired-pulse ratio (PPR) was calculated by determining the ratio of the peak amplitude of the eEPSC2/eEPSC1 induced by the pairs of stimuli, triggered at a 50-ms inter-pulse interval. Furthermore, paired-pulse stimulations with a 50 ms inter-pulse interval were given during the pre-LTP recording. For pre-LTP induction, 240 paired presynaptic stimuli (with 50 ms inter-pulse intervals) were delivered at 2 Hz to the presynaptic fibers at a holding potential of −60 mV. The ratio of currents mediated by AMPAR and NMDAR was assessed in the presence of 100 μM PTX at a holding membrane potential of −70 mV and +40 mV, respectively. The AMPAR/NMDAR ratio was determined as the quotient of the average peak EPSC amplitude at −70 mV to the average EPSC amplitude recorded at +40 mV (averaged at 40 ms after afferent stimulation). All EPSCs used for analyses were averaged from 10 consecutive traces. For post-LTP induction, a pairing LTP protocol was used by delivering 80 pulses at 2 Hz paired with postsynaptic depolarization at +30 mV after obtaining stable EPSCs for 10 min. Summary LTP graphs were plotted by normalizing data in five-minute epochs against the baseline EPSCs mean.

### Statistical analysis

Sample sizes and statistical methodologies are detailed in the figure legends. Required sample sizes were determined based on previous studies [9, 18, 19], without employing statistical methods for predetermination. Statistical analyses and graphical representations were executed using GraphPad Prism 9 (GraphPad Software, Inc., USA) and SPSS v. 20.0. Data are expressed as the *mean* *±* *SEM*. Normality was assessed via the *Shapiro-Wilk* test, and homogeneity of variance was evaluated using *Levene*’s test. Data conforming to these two conditions were analyzed with a two-tailed unpaired *t*-test for comparisons between two independent groups or two-way repeated-measures (RM) ANOVA, followed by *Tukey*’s or *Sidak*’s multiple comparisons test for multivariable comparisons involving multiple groups or variables. In case of absence of distribution normality, non-parametric tests, such as the Mann-Whitney U test or the Scheirer-Ray-Hare test (a two-factor non-parametric analysis), were applied, followed by *Dunn*’s multiple comparisons test. A *p*-value of less than 0.05 was considered statistically significant, with a confidence level set at 95%.

## Results

### TaVNS significantly alleviates anxiety-like behaviors in PTSD-like mice

First, we assessed the well-being of these mice by monitoring body weight, body temperature, and feed intake throughout the taVNS period. The findings revealed no significant differences in these parameters across the four groups based on the well-being assessments (Fig. S1).

**Fig. 1:**
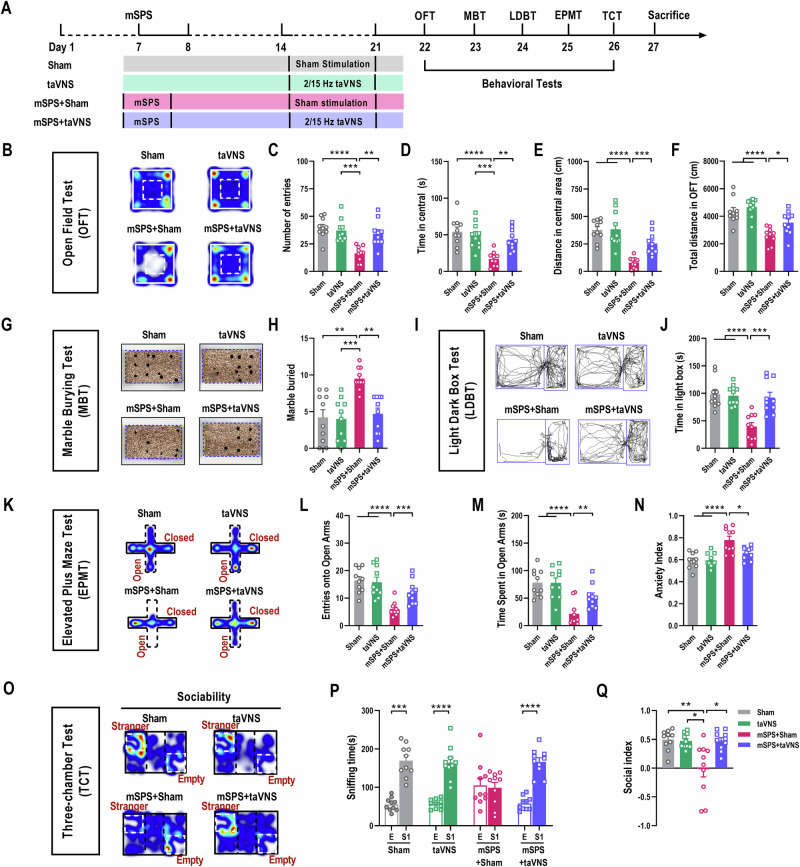
TaVNS significantly alleviated mSPS-induced anxiety-like behaviors and social interaction deficits. **(A)** Experimental scheme. (**B)** Representative traces during OFT in Sham, taVNS, mSPS+Sham, and mSPS+taVNS mice. **(C-F)** Entry times **(C)**, duration **(D)**, traveled distance in the central area **(E)**, and total distance **(F)** in the open field of four groups [Two-way RM ANOVA with *Tukey*’s multiple comparisons test: **(C)** F_(1, 36)_ = 11.38, *p* = 0.0018; Sham *vs* mSPS+Sham, *p* < 0.0001; taVNS *vs* mSPS+Sham, *p* = 0.0002; mSPS+Sham *vs* mSPS+taVNS, *p* = 0.0011. **(D)** F_(1, 36)_ = 10.14, *p* = 0.0030; Sham *vs* mSPS+Sham, *p* < 0.0001; taVNS *vs* mSPS+Sham, *p* = 0.0004; mSPS+Sham *vs* mSPS+taVNS, *p* = 0.0034. **(E)** F_(1, 36)_ = 12.32, *p* = 0.0012; Sham *vs* mSPS+Sham, *p* < 0.0001; taVNS *vs* mSPS+Sham, *p* < 0.0001; mSPS+Sham *vs* mSPS+taVNS*, p* = 0.0001. **(F)** F_(1, 36)_ = 2.4540, *p* = 0.1260; Sham *vs* mSPS**+**Sham, *p* < 0.0001; taVNS *vs* mSPS+Sham, *p* < 0.0001; mSPS+Sham *vs* mSPS+taVNS, *p* = 0.0236]. **(G)** Representative photograph after MBT among the four groups. **(H)** The number of marbles buried in four groups [Scheirer-Ray-Hare test followed by non-parametric *Dunn*’s multiple comparisons test: H = 4.8651, *p* = 0.0274; Sham *vs* mSPS+Sham, *p* = 0.0019; taVNS *vs* mSPS+Sham, *p* = 0.0006; mSPS+Sham *vs* mSPS+taVNS, *p* = 0.0037]. **(I)** Representative traces during LDBT among the four groups. **(J)** Time spent in the light box [Two-way RM ANOVA with *Tukey*’s multiple comparisons test: F_(1, 36)_ = 12.35, *p* = 0.0012; Sham *vs* mSPS+Sham, *p* < 0.0001; taVNS *vs* mSPS+Sham, *p* < 0.0001; mSPS+Sham *vs* mSPS+taVNS, *p* = 0.0002]. **(K)** Representative traces during EPMT among the four groups. **(L-N)** Entry times **(L)** and duration **(M)** in the open arms, as well as the anxiety index **(N)** of four groups **[**Two**-**way RM ANOVA with *Tukey*’s multiple comparisons test: **(L)** F_(1, 36)_ = 12.45, *p* = 0.0012; Sham *vs* mSPS+Sham, *p* < 0.0001; taVNS *vs* mSPS+Sham, *p* < 0.0001; mSPS+Sham *vs* mSPS+taVNS, *p* = 0.0003. **(M)** F_(1, 36)_ = 7.953, *p* = 0.0078; Sham *vs* mSPS+Sham, *p* < 0.0001; taVNS *vs* mSPS+Sham, *p* < 0.0001; mSPS+Sham *vs* mSPS+taVNS, *p* = 0.0021. **(N)** F_(1, 36)_ = 4.955, *p* = 0.0342; Sham *vs* mSPS+Sham, *p* < 0.0001; taVNS *vs* mSPS+Sham, *p* < 0.0001; mSPS+Sham *vs* mSPS+taVNS, *p* = 0.0247**]. (O)** Representative traces during TCT among the four groups. **(P, Q)** Sniffing time **(P)** and social index **(Q)** of four groups [**(P)** Mann**-**Whitney U test: Sham group, E *vs* S1, Z = −3.7796, *p* < 0.0001; Two-tailed unpaired *t***-**test**:** taVNS group, E *vs* S1, *t*_(18)_ = 7.5720, *p* < 0.0001; mSPS**+**Sham group, E *vs* S1, *t*_(18)_ = 0.2654, *p* = 0.7937; mSPS+taVNS group, E *vs* S1, *t*_(18)_ = 8.9290, *p* < 0.0001; **(Q)** Scheirer-Ray-Hare test followed by non-parametric *Dunn*’s multiple comparisons test: H = 5.2866, *p* < 0.0001; Sham *vs* mSPS+Sham, *p* = 0.0096; taVNS *vs* mSPS+Sham, *p* = 0.0374; mSPS+Sham *vs* mSPS+taVNS, *p* = 0.0279]. n = 10 mice per group. Data represent the *mean* *±* *SEM*. ^*^p < 0.05, ^**^p < 0.01, ^***^p < 0.001, ^****^p < 0.0001.

In controlled laboratory settings, anxiety-like behaviors in mice are often assessed using the OFT, LDBT, and EPMT to measure avoidance of bright and open spaces, MBT to measure repetitive stereotyped behavior, as well as TCT to measure social avoidance. These behavioral features are commonly utilized to gauge the anxiety state [24, 25]. To assess whether these mice display anxiety-like behaviors after exposure to the mSPS procedure and taVNS treatments, we used open-field, marble-bead, light-dark box, elevated plus maze, and the three-chamber assays to measure anxiety levels (Fig. 1A). In the OFT, Sham and taVNS groups extensively explored the central area, whereas mSPS+Sham mice entered the central area less often, spent less time, and traveled less distance in the central area (Fig. 1B–E). Additionally, the total traveled distance of the mSPS+Sham mice were also significantly decreased, indicating a reduction in spontaneous and exploration behaviors (Fig. 1F). Moreover, in the MBT and LDBT, the mSPS+Sham group showed increased total numbers of buried marbles (Fig. 1G and H) and reduced duration staying in the light box (Fig. 1I and J) relative to Sham and taVNS groups, respectively. In the EPMT, mSPS+Sham mice preferred to stay in the safely closed arms, showed fewer entries and less duration exploring the open arm, as well as had a higher anxiety index compared with Sham and taVNS groups (Fig. 1K–N). In the TCT, compared with the Sham and taVNS groups, the mSPS+Sham group spent less time in the compartment containing the stranger mouse and showed a lower social index (Fig. 1O–Q). These results indicate that only those exposed to isoflurane with sham stimulation or taVNS cannot induce negative effects on the tested behaviors in C57 BL/6 J mice. In contrast, mSPS+Sham mice exhibited significant anxiety-like behaviors [26].

Then, we observed the effect of taVNS on mSPS-triggered anxiety-like behaviors. Compared with the mSPS+Sham group, mSPS+taVNS mice markedly increased entering times, duration of stay, and traveled distance in the OFT (Fig. 1B–F). At the same time, they also reduced the abnormal marble-burying behaviors (Fig. 1G, H) and showed more duration staying in the light box (Fig. 1I, J). Moreover, they spent a significant amount of time exploring the open arms and exhibited a lower anxiety index (Fig. 1K–N). And they also interacted more with stranger mice and exhibited a relatively higher social index (Fig. 1O–Q). These results demonstrated that taVNS could improve reduced spontaneous behaviors and increased anxiety-like behaviors in mSPS-exposed mice.

Locomotor activity is strongly linked to maladaptive changes in animal models of PTSD [27]. Several studies have consistently documented that exposure to SPS or foot shocks decreases locomotor activity in rodent animals [28, 29]. This reduced exploration of novel environments is indicative of stress hyper-responsiveness and fear generalization [28]. To further elucidate that the observed alterations in locomotor activity among these mice are not attributable to motor deficits, we employed the CatWalk XT gait analysis system to characterize their motor behaviors. The results indicated that neither exposure to mSPS nor taVNS treatments was sufficient to induce any notable changes in gait (Fig. S2).

Considering the influence of VNS on pain regulation, which may involve both inhibitory and facilitatory modulation of pain [30], we sought to determine whether the effects of taVNS on anxiety-like behaviors could be ascribed to alterations in the pain responses of the PTSD-like mice. To investigate this, we compared sensitivity to mechanical and thermal stimuli across the Sham, taVNS, mSPS+Sham, and mSPS+taVNS groups within our laboratory experimental setting to exclude any potential impact of pain response on anxiety-like behaviors. The results showed no significant differences in the responses to mechanical or thermal stimuli among the four groups (Fig. S3). Based on these findings, the possibility that the taVNS effects on anxiety-like behaviors in PTSD-like mice are mediated through changes in their motor ability and pain responses can be excluded.

### Genetic capture of taVNS-activated neurons in the ACC

To elucidate the brain regions potentially contributing to the effects of taVNS in PTSD-like mice, we utilized a reporter line derived from crossing FosTRAP2 mice with the Rosa26-TdTomato reporter line to identify neurons activated during taVNS treatments (Fig. S4). Comparative analyses of neuronal activation in sham- and taVNS-treated PTSD-like mice revealed significant differences in the number of TRAP/TdTomato-positive (TRAP/TdTomato^+^) cells across multiple brain regions, including the prefrontal cortex (PFC), ACC, caudate putamen (CPu), nucleus accumbens (NAc), lateral septal nucleus (LSI), retrosplenial cortex (RSG), lateral habenular nucleus (LHb), mediodorsal thalamus (MD), medial preoptic nucleus (MPO), paraventricular thalamic nucleus (PVA), ventral posteromedial thalamic nucleus (VPM), basolateral amygdala (BLA), periaqueductal gray (PAG), lateral parabrachial nucleus (LPB) and nucleus of the solitary tract (NTS). Notably, the ACC and CPu exhibited the largest fold increases in TRAP/TdTomato^+^ cell counts in the PTSD+taVNS group relative to the PTSD+sham group (Fig. S4). Furthermore, the ACC displayed a significantly greater number of TRAP/TdTomato^+^ cells than the CPu in PTSD+taVNS mice. Given the ACC’s well-established role in regulating emotional and behavioral responses, particularly in modulating stress-induced negative effects [8–13], we propose that taVNS-induced activation of ACC neurons constitutes a critical mechanism for alleviating anxiety-like behaviors.

**Fig. 2:**
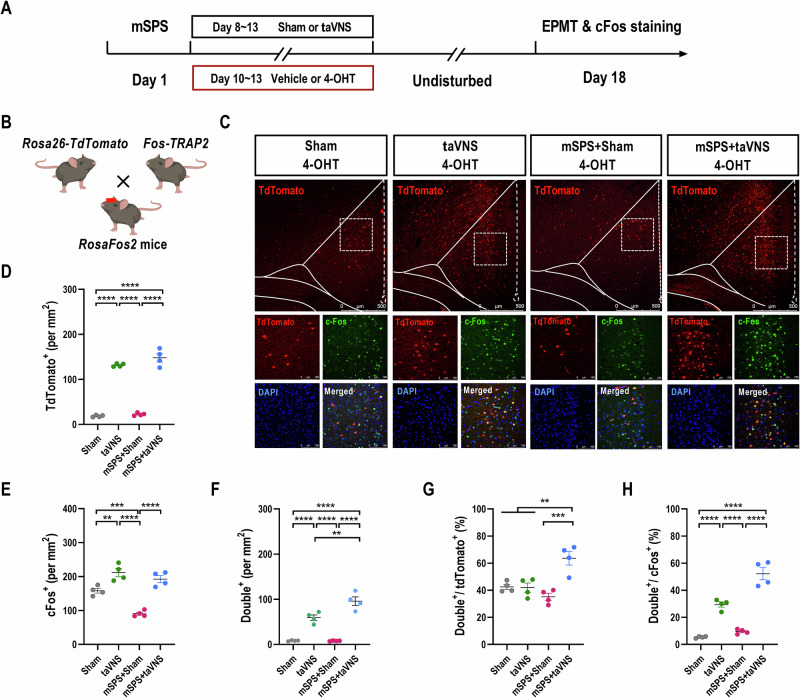
Captured TANs in the ACC of PTSD-like mice with 4-OHT injection, which is reactivated upon the EPMT. **(A)** Experimental scheme. **(B)** Diagram of FosTRAP2 mice crossed with Rosa26-tdTomato mice (Created with BioRender.com, 2025). **(C)** Top: Representative images of *fos*-trapped neurons expressing tdTomato in the ACC from RosaFos2 mice among the Sham, taVNS, mSPS+Sham, and mSPS+taVNS groups. Scale bar, 500 μm. Bottom: Co-localization of TANs and cFos induction following the EPMT in the ACC. Scale bar, 100 μm. **(D)** The number of tdTomato^+^ cells [Two-way RM ANOVA with *Tukey*’s multiple comparisons tests: F_(1, 12)_ = 0.5792, *p* = 0.4613; Sham *vs* taVNS, *p* < 0.0001; Sham *vs* mSPS+taVNS, *p* < 0.0001; taVNS *vs* mSPS+Sham, *p* < 0.0001; mSPS+Sham *vs* mSPS+taVNS, *p* < 0.0001]. **(E)** The number of cFos^+^ cells [Two-way RM ANOVA with *Tukey*’s multiple comparisons tests: F_(1, 12)_ = 7.312, *p* = 0.0192; Sham *vs* taVNS, *p* = 0.0053; Sham *vs* mSPS+Sham, *p* = 0.0009; taVNS *vs* mSPS+Sham, *p* < 0.0001; mSPS+Sham *vs* mSPS+taVNS, *p* < 0.0001]. **(F)** The number of cFos^+^ & tdTomato^+^ double-positive cells [Two-way RM ANOVA with *Tukey*’s multiple comparisons tests: F_(1, 12)_ = 10.06, *p* = 0.0080; Sham *vs* taVNS, *p* < 0.0001; Sham *vs* mSPS+taVNS, *p* < 0.0001; taVNS *vs* mSPS+Sham, *p* < 0.0001; taVNS *vs* mSPS+taVNS, *p* = 0.0077; mSPS+Sham *vs* mSPS+taVNS, *p* < 0.0001]. **(G)** The number of double-positive cells is normalized to the total number of tdTomato^+^ cells. [Two-way RM ANOVA with *Tukey*’s multiple comparisons tests: F_(1, 12)_ = 17.88, *p* = 0.0012; Sham *vs* mSPS^+^taVNS, *p* = 0.0045; taVNS *vs* mSPS+taVNS, *p* = 0.0037; mSPS+Sham *vs* mSPS+taVNS, *p* = 0.0004]. **(H)** The number of double-positive cells is normalized to the total number of cFos^+^ cells. [Two-way RM ANOVA with *Tukey*’s multiple comparisons tests: F_(1, 12)_ = 6.6990, *p* = 0.0237; Sham *vs* taVNS, *p* < 0.0001; Sham *vs* mSPS+taVNS, *p* < 0.0001; taVNS *vs* mSPS+Sham, *p* < 0.0001; mSPS+Sham *vs* mSPS+taVNS, *p* < 0.0001]. n = 4 mice per group. Data represent the *mean* *±* *SEM*. ^*^p < 0.05, ^**^p < 0.01, ^***^p < 0.001, ^****^p < 0.0001.

To further investigate whether ACC neurons engaged in communicating with signals triggered by taVNS, and to assess the reactivation extent of taVNS-activated neurons (TANs) during anxiety-like behavioral tests, we trapped TANs in the ACC of PTSD-like mice using the Fos-TRAP and immunohistochemistry approach (Fig. 2A, B) [31]. Following exposure to the mSPS procedure, vehicle or 4-OHT was injected into the RosaFos2 mice immediately after sham stimulation or taVNS treatments (Fig. 2 and Fig. S5). To evaluate the effects of sham stimulation and taVNS, mice were sacrificed one hour later to stain the immediate-early gene cellular oncogene (cFOS) following the EPMT. The findings implied that neurons activated by 4-OHT (Fig. 2C), but not by the vehicle (Fig. S5A), during sham stimulation or taVNS, exhibited TRAP/tdTomato^+^ expression in the ACC of RosaFos2 mice. Furthermore, a higher density of tdTomato^+^ neurons was captured in the ACC of normal and PTSD-like mice that received taVNS compared to sham stimulation (Fig. 2C and D). This confirms the presence of TANs, indicating that treatments with taVNS alone are sufficient to activate neurons in the ACC. Importantly, following the EPMT, the levels of cFos expression were significantly decreased in the mSPS+Sham group compared to the Sham and taVNS groups. In contrast, this reduction in cFos expression was reversed in mSPS mice after taVNS treatments (Fig. 2C and E, Fig. S5B). Moreover, a higher proportion of tdTomato^+^ neurons in the mSPS+taVNS group was correlated with an increased density of cFos^+^ neurons (Fig. 2F–H). This pattern of results indicates that neuronal activity in the ACC during anxiety tests is highly selective for TANs that were tagged during taVNS treatments.

### Characterizing the neuronal type of TANs^ACC^

**Fig. 3:**
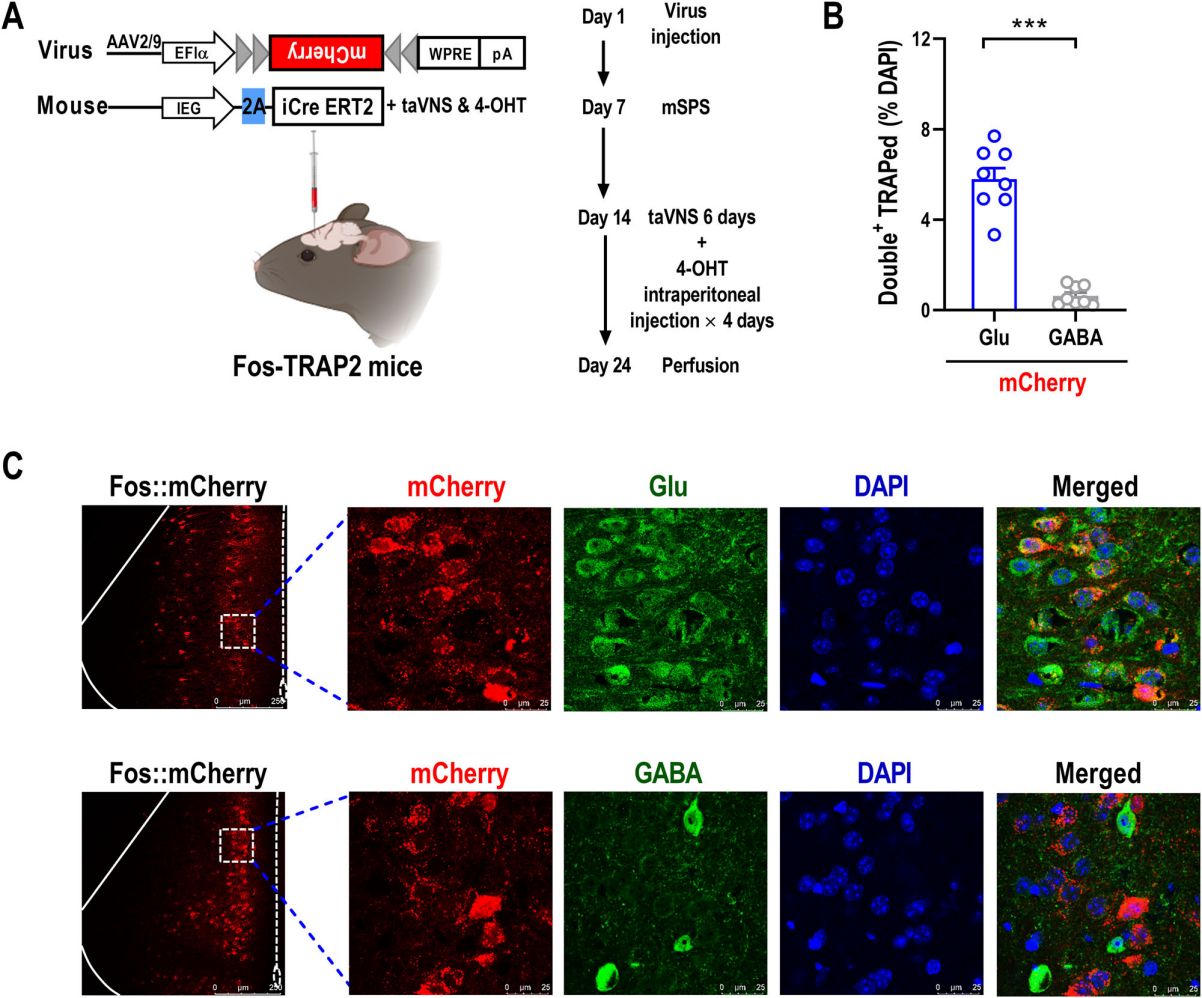
Glutamatergic TANs^ACC^ are significantly greater than that of GABAergic neurons in PTSD-like mice. **(A)** Schematic illustration of the Fos-TRAP strategy used to label TANs^ACC^ (Created with BioRender.com, 2025). **(B)** Statistical analysis shows that mCherry-­labeled neurons within the ACC co-localize with glutamate (Glu) and GABA antibodies, quantified as a percentage of the total DAPI-positive cell population [n = 8 mice per group. Mann-Whitney U test: *Z* = −3.3607, *p* = 0.0002]. **(C)** Representative images illustrating the infusion site within the ACC (left) and mCherry-­labeled neurons co-localizing with Glu (upper) or GABA (bottom) antibody (right). Scale bar, 250 μm (left) and 100 μm (right). Data represent the *mean* *±* *SEM*. ^*^p < 0.05, ^**^p < 0.01, ^***^p < 0.001.

To identify the cell-typed signatures of TANs^ACC^, we evaluated the colocalization of TRAP/tdTomato^+^ neurons with two types of ACC neurons: the glutamatergic and GABAergic neurons. To achieve this, we injected AAV-EFlα-DIO-mCherry into the ACC of Fos-TRAP2 mice, applying the same induction paradigm as previously described (Fig. 3A). Following exposure to the mSPS procedure and subsequent taVNS treatment, immunofluorescent staining showed that the proportion of TANs^ACC^ co-localizing with glutamate (Glu) or γ-aminobutyric acid (GABA) represented 5.786% ± 0.4985 or 0.6276% ± 0.1486 of the total DAPI-positive cell population, respectively (Fig. 3B and C). These findings demonstrate that the proportion of glutamatergic TANs^ACC^ is significantly greater than that of GABAergic TAN^ACC^ during taVNS, potentially contributing to the alleviation of anxiety-like behaviors in PTSD-like mice.

### TaVNS enhances presynaptic excitatory transmission of the glutamatergic TANs^ACC^ in PTSD-like mice

**Fig. 4:**
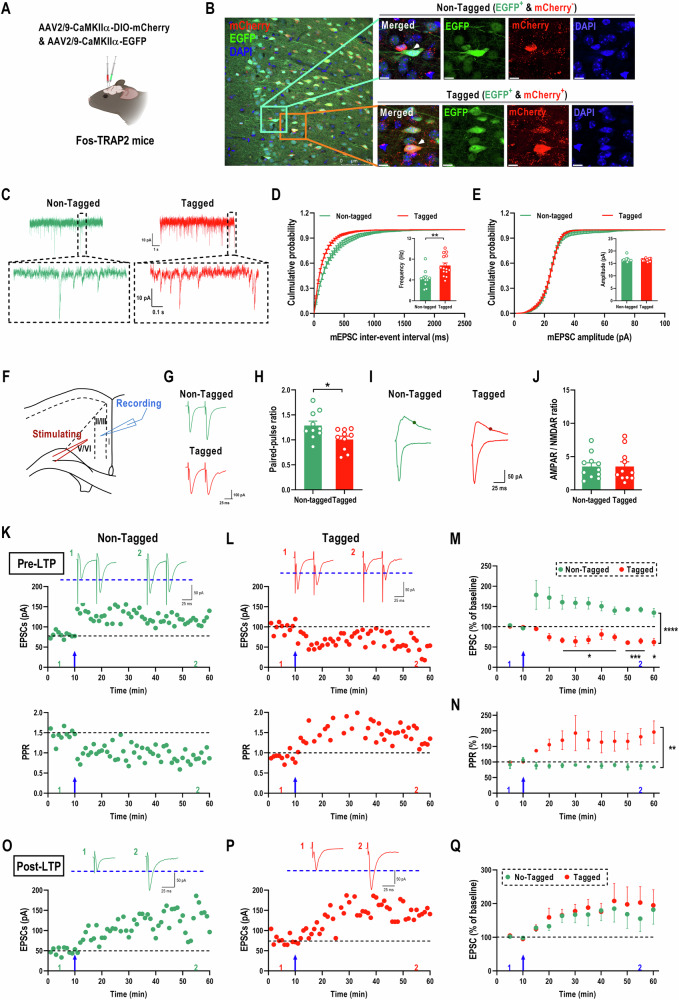
TaVNS treatments enhanced the presynaptic glutamate transmission in glutamatergic TANs^ACC^ (Tagged) neurons of PTSD-like mice. **(A)** Schematic of AAV-CaMKIIα-DIO-mCherry and AAV-CaMKIIα-EGFP mixture injection into the ACC of FosTRAP2 mice (Created with BioRender.com, 2025). **(B)** Representative images showing non-tagged neurons only expressed EGFP, as well as tagged neurons labeled with both EGFP and mCherry within the ACC, scale bar, 75 µm (left) and 10 μm (right). **(C)** Representative traces of mEPSC recording from non-tagged and tagged neurons. **(D)** Summary data for mEPSC frequency (right) with cumulative probability plots of inter-event intervals (left) in non-tagged and tagged neurons [Non-tagged neurons: n = 11 cells from 5 mice; Tagged neurons: n = 15 cells from 5 mice. Two-tailed unpaired *t*-test for mEPSC frequency: *t*_(24)_ = 3.5040, *p* = 0.0018]. **(E)** Summary data for mEPSC amplitude (right) with cumulative probability plots of amplitude (left) in non-tagged and tagged neurons. [Non-tagged neurons: n = 11 cells from 5 mice; Tagged neurons: n = 15 cells from 5 mice. Mann-Whitney U test for mEPSC amplitude (left): *Z* = −0.1298, *p* = 0.9188]. **(F)** Schematic diagram showing the placement of stimulating and recording electrodes in the ACC (Created with BioRender.com, 2025). **(G)** Representative recording traces of PPR from non-tagged and tagged neurons. **(H)** Summary data for PPR in non-tagged and tagged neurons [Non-tagged neurons: n = 10 cells from 5 mice; Tagged neurons: n = 10 cells from 5 mice. Two-tailed unpaired *t*-test: *t*_(18)_ = 2.541, *p* = 0.0205]. **(I)** Representative recording traces of the AMPAR/NMDAR ratio from non-tagged and tagged neurons. **(J)** Summary data for the ratio of AMPAR- and NMDAR-mediated currents in non-tagged and tagged neurons [Non-tagged neurons: n = 11 cells from 5 mice; Tagged neurons: n = 11 cells from 5 mice. Mann-Whitney U test: *Z* = 0.2955, *p* = 0.7969]. **(K, L)** Top: sample traces of eEPSCs with paired-pulse stimulation at 50 ms inter-stimulus interval during baseline (1) and last 10 min (2) at a holding membrane potential of −60 mV. Middle: a time course plot of a representative single example. Bottom: time course plot of PPR for this neuron. The arrow denotes the time of pre-LTP induction. These data were recorded from non-tagged **(K)** and tagged **(L)** neurons. **(M)** Pooled data to illustrate the time course of pre-LTP recording from non-tagged and tagged neurons [Non-tagged: n = 4 cells from 3 mice; Tagged: n = 5 cells from 3 mice. Mixed-effects model analysis with the Geisser-Greenhouse correction, coupled with *Sidak*’s multiple comparisons test: F_(11, 77)_ = 7.7800, *p* < 0.0001]. (**N**) Pooled data to illustrate the time course of changes in PPR for these neurons [Non-tagged neurons: n = 4 cells from 3 mice; tagged neurons: n = 5 cells from 3 mice. Mixed-effects model analysis with the Geisser-Greenhouse correction: F_(11, 77)_ = 2.6380, *p* = 0.0065]. (O-P) Top**:** sample traces of eEPSCs from post-LTP recordings during baseline (1) and last 10 min (2) at a holding membrane potential of −60 mV. Bottom: time course plot of eEPSCs for this neuron. The arrow denotes the time of post-LTP induction. These data were recorded from non-tagged (**O**) and tagged (**P**) neurons**. (Q**) Pooled data to illustrate the time course of post-LTP recording from non-tagged and tagged neurons [Non-tagged: n = 5 cells from 4 mice; Tagged: n = 4 cells from 4 mice. Mixed-effects model analysis with the Geisser-Greenhouse correction: F_(1, 77)_ = 0.3511, *p* = 0.9704]. Data represent the *mean* *±* *SEM*. ^*^p < 0.05, ^**^p < 0.01, ^***^p < 0.001, and ^****^p < 0.0001.

There is a hypothesis that neurons activated by taVNS become functionally different from non-activated neurons due to plasticity. Therefore, it is important to investigate whether the glutamatergic TANs^ACC^ (tagged neurons) display different basal synaptic transmission properties compared to those non-tagged glutamatergic neurons in the ACC, which may explain the long-lasting positive effects of taVNS. To explore this possibility, we used an activity-dependent fluorescence tag along with the constitutive labeling of the glutamatergic neurons in the ACC, injecting AAV-CaMKIIα-EGFP and AAV-CaMKIIα-DIO-mCherry (ratio 1.5:1) mixed vectors into the bilateral ACC of Fos-TRAP2 mice that would undergo the mSPS procedure and taVNS treatments followed by 4-OHT injection (Fig. 4A).

Given the necessary relationship between the activity of the glutamatergic TANs^ACC^ and the effects of taVNS in PTSD-like mice, we recorded mEPSC on tagged neurons, which were labeled with both EGFP and mCherry, as well as non-tagged neurons, which only contained EGFP, from the same slices (Fig. 4B). The results indicated that the mEPSC frequency in the tagged group was higher than that in the non-tagged group (Fig. 4C and D), however, there were no differences observed in the mEPSC peak amplitude between two groups (Fig. 4C and E). To further confirm this, we measured the PPR on tagged and non-tagged neurons in the ACC to evaluate presynaptic release probability (Fig. 4F). We observed a marked reduction in PPR on tagged neurons compared to non-tagged neurons (Fig. 4G and H), implying enhanced presynaptic release probabilities of the glutamatergic presynaptic terminals in innervating tagged neurons in mSPS+taVNS mice. Then, to determine whether taVNS treatments affect glutamatergic transmission at the postsynaptic receptor level, the ratio of the amplitudes of evoked AMPAR- and NMDAR-EPSCs (AMPAR/NMDAR ratio) in tagged and non-tagged neurons were recorded. Consistent with the results from the amplitude of mEPSC in these two types of neurons in the ACC, there were no significant differences in the AMPAR/NMDAR ratio on tagged neurons compared with the non-tagged neurons (Fig. 4I and J). This implies that taVNS enhanced the level of basal transmission on the glutamatergic TANs^ACC^ by increasing the presynaptic release of glutamate, but not on those non-activated glutamatergic neurons of ACC.

### The potentiation of presynaptic release of glutamate prevents further plastic changes in the glutamatergic TANs^ACC^

To further explore the fundamental mechanisms underlying the enhancement of presynaptic release probabilities, we implemented a low-frequency stimulating protocol (2 Hz for 2 min) to induce pre-LTP in the ACC [9, 32]. The results revealed a significant increase in the amplitude of eEPSC in non-tagged neurons, where the LTP was sustained for a minimum of 50 min (141.38% of baseline; Fig. 4K and M). Conversely, the tagged neurons, which had previously exhibited enhanced presynaptic release probabilities, displayed a decreased amplitude of eEPSC (67.60% of baseline; Fig. 4L and M). Additionally, the potentiation of EPSC amplitude in non-tagged neurons of the ACC corresponded with a reduction in the PPR (87.26% of baseline, Fig. 4N), which is commonly applied to evaluate presynaptic function. In contrast, the tagged neurons showed an enhancement in the PPR (166.29% of baseline, Fig. 4N).

Next, we examined whether taVNS treatments also affect post-LTP in the tagged and non-tagged neurons of ACC and found that post-LTP exhibited no significant differences (Fig. 4O–Q). These results indicate that after exposure to mSPS, tagged neurons exhibited improved presynaptic excitatory transmissions following taVNS treatments, thereby preventing further synaptic potentiation, expressed as depotentiation, within the same tagged population.

### Selective inhibition of glutamatergic TANs^ACC^ weakens the positive effects of taVNS on PTSD-related anxiety-like behaviors

**Fig. 5:**
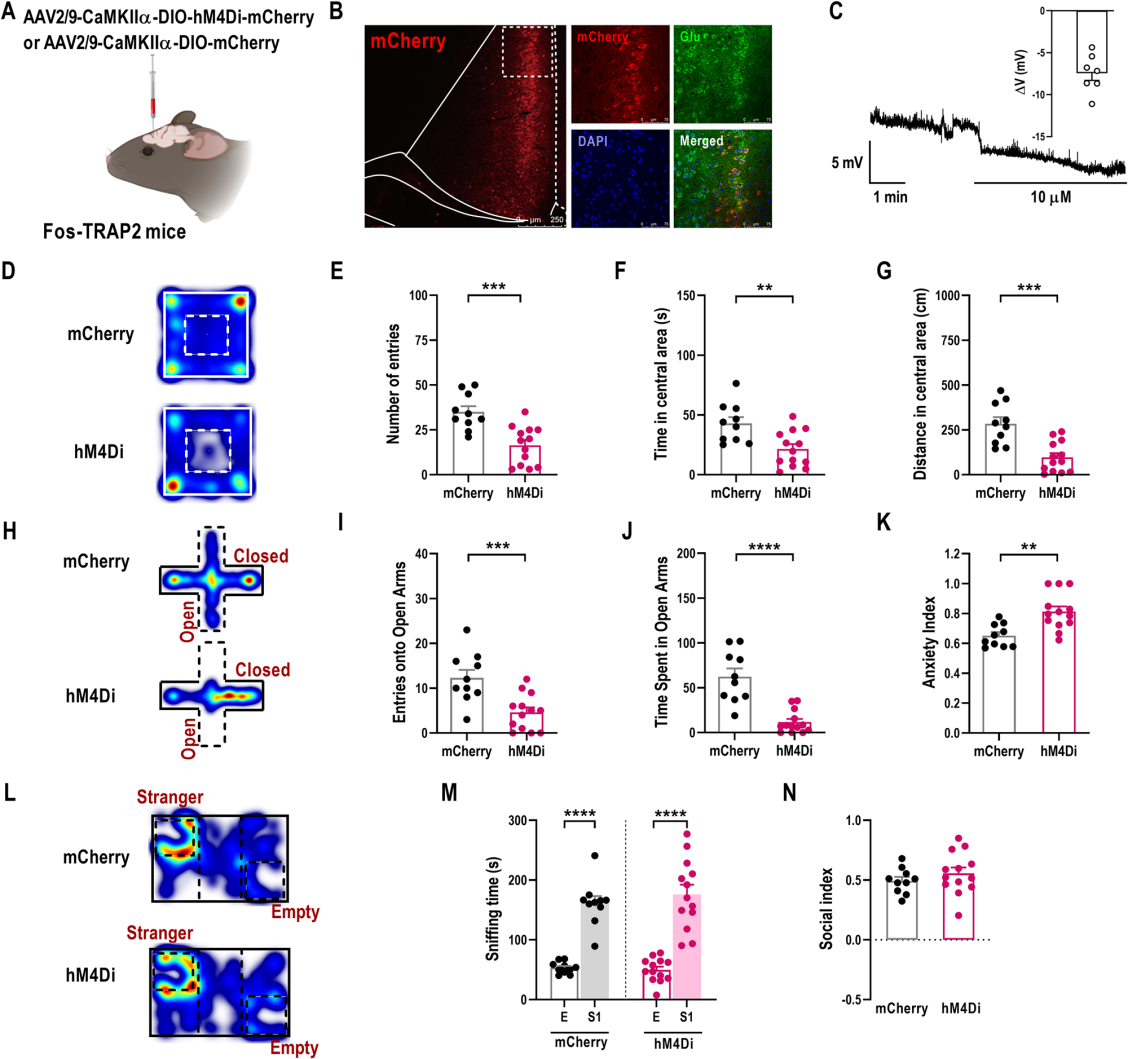
Selective inhibition of the activity of glutamatergic TANs^ACC^ neurons weakened the positive effects of taVNS on PTSD-related anxiety-like behaviors. **(A)** Schematic of AAV-CaMKIIα-DIO-hM4Di-mCherry or AAV-CaMKIIα-DIO-mCherry injection into the ACC of Fos-TRAP2 mice (Created with BioRender.com, 2025). **(B)** Representative images showing the infusion site within the ACC (left) and mCherry­labeled neurons co-expressed with glutamate (Glu) antibody (right), scale bar, 250 μm (left), and 75 μm (right). **(C)** Representative trace (left) and summarized data (right) showing bath application of CNO (10 μM) hyperpolarizes the glutamatergic TANs^ACC^ neurons (n = 7 neurons from 3 mice). **(D)** Representative traces during OFT in mCherry and hM4Di mice. **(E-G)** Entry times **(E)**, duration **(F)**, and traveled distance **(G)** in the central area of the open field of two groups [Two-tailed unpaired *t*-test: **(E)** *t*_(21)_ = 4.2590, *p* = 0.0003. **(F)** *t*_(21)_ = 3.2700, *p* = 0.0037. **(G)** *t*_(21)_ = 4.480, *p* = 0.0002]. **(H)** Representative traces during EPMT in two groups. **(I-K)** Entry times **(I)**, and duration **(J)** in the open arms, as well as anxiety index **(K)** of two groups [Two-tailed unpaired *t*-test: **(I)** *t*_(21)_ = 3.841, *p* = 0.0010. **(K)** *t*_(21)_ = 3.6550, *p* = 0.0015. Mann-Whitney U test: **(J)** *Z* = −2.9860, *p* < 0.0001]. (**L**) Representative traces during TCT in two groups. (**M**, **N**) Sniffing time (**M**), and social index (**N**) of two groups [(**M**) Two-tailed unpaired t-test: mCherry: E *vs* S1, *t*_(18)_ = 8.1850, *p* < 0.0001; Mann-Whitney U test: hM4Di: E *vs* S1, Z = −4.3333, *p* < 0.0001. (**N**) Two-tailed unpaired *t*-test: *t*_(21**)**_ = 1.020, *p* = 0.3193]. mCherry_:_ n = 10 mice, hM4Di: n = 13. Data are represented as *mean* *±* *SEM*. ^*^p < 0.05, ^**^p < 0.01, ^***^p < 0.001, ^****^p < 0.0001.

The modification of basal synaptic transmission likely influences neuronal activity, which is essential for emotional behaviors [19]. To directly investigate whether the activity of glutamatergic TANs^ACC^ is the target of taVNS, we employed a Gi-coupled chemogenetic approach to inhibit these neurons in Fos-TRAP2 mice (Fig. 5A). Specifically, AAV-CaMKIIα-DIO-hM4Di-mCherry was bilaterally injected into ACC, enabling the expression of hM4Di receptors selectively in glutamatergic TANs^ACC^ (Fig. 5B). Following sufficient expression, we carried out whole-cell recordings to confirm a reduction in neuronal excitability of CaMKIIα-hM4Di-mCherry-positive glutamatergic TANs^ACC^ by bath application of 10 µM CNO (Fig. 5C). Then, we tested the effects of 5 mg/kg CNO on anxiety-like behaviors in the mSPS+taVNS group. In parallel, control mice subjected to mSPS exposure and taVNS interventions, expressing mCherry alone (AAV-CaMKIIα-DIO-mCherry), were administered equivalent CNO dosages and underwent behavioral assessments. As shown in Fig. 5D–G, when CNO was injected for forty minutes to suppress the activity of glutamatergic TANs^ACC^ expressed hM4Di receptors, the mice made fewer entries, spent less time, and traveled shorter distances in the central area of OFT. At the same time, they reduced the entries and duration of stay in the open arms of EPMT (Fig. 5H–J), showing a relatively higher anxiety index (Fig. 5K), compared to the mCherry group. Notably, the hM4Di mice exhibited unaffected sociability, which means that specifically inhibiting the activity of glutamatergic TANs^ACC^ had a negligible effect on their social interaction (Fig. 5L–N). These findings suggest that taVNS primarily modulates the activation of glutamatergic TANs^ACC^ neurons to ameliorate anxiety-like emotional behaviors in mice with PTSD, but may not have social aspects.

### Selective activation of glutamatergic TANs^ACC^ does not further amplify the positive effects of taVNS

**Fig. 6:**
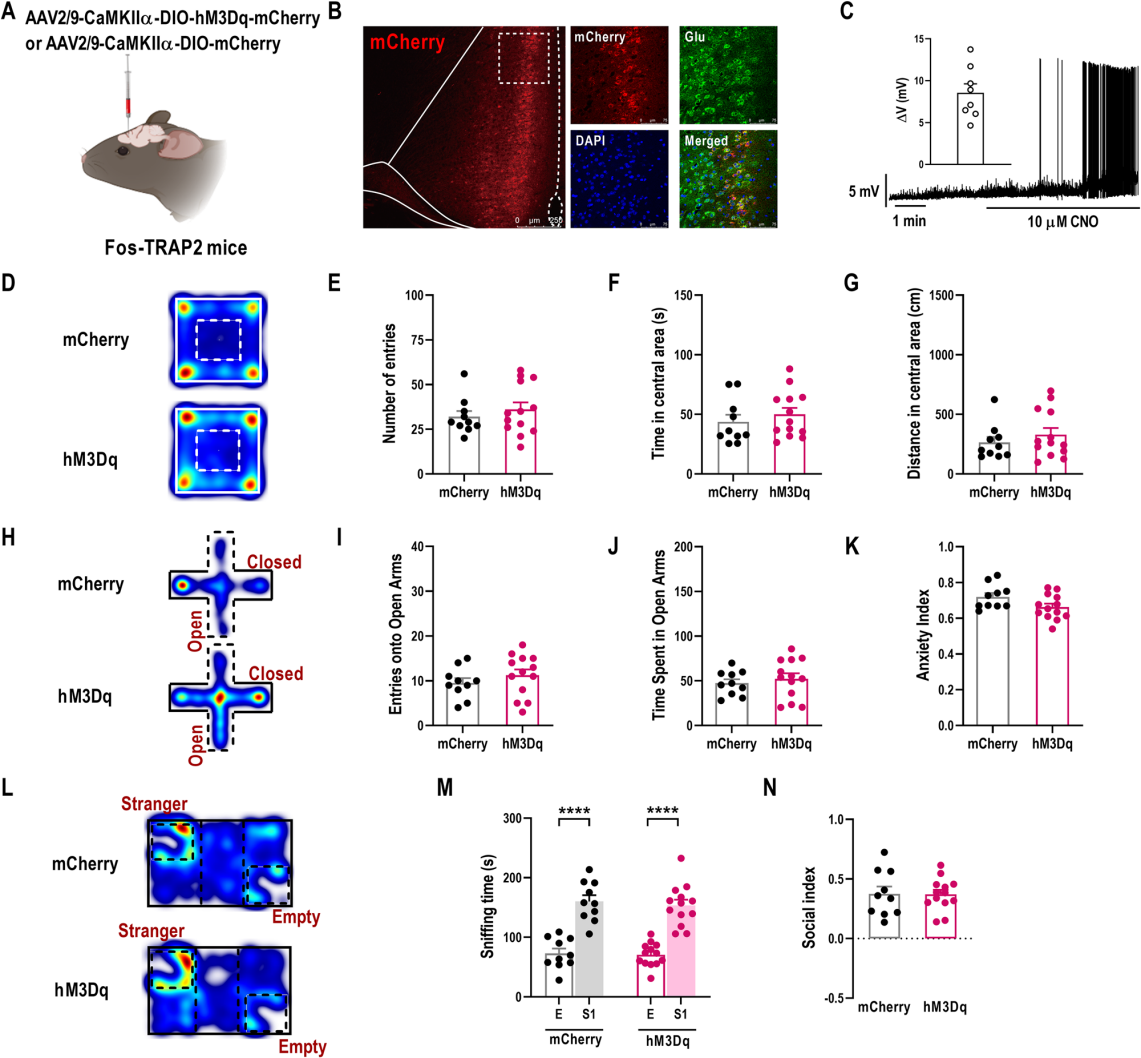
Selective activation of glutamatergic TANs^ACC^ neurons does not further amplify the positive effects of taVNS on PTSD-related anxiety-like behaviors. **(A)** Schematic of AAV-CaMKIIα-DIO-hM3Dq-mCherry or AAV-CaMKIIα-DIO-mCherry injection into the ACC of Fos-TRAP2 mice (Created with BioRender.com, 2025). **(B)** Representative images showing the infusion site within the ACC (left) and mCherry­labeled neurons co-expressed with glutamate antibody (right). Scale bar, 250 μm (left) and 75 μm (right). **(C)** Summarized data (left) and representative trace (right) showing bath application of CNO (10 μM) depolarizes the glutamatergic TANs^ACC^ neurons (n = 8 neurons from 3 mice). **(D)** Representative traces during OFT in mCherry and hM3Dq mice. **(E-G)** Entry times **(E)**, duration **(F)**, and traveled distance **(G)** in the central area of the open field of two groups in the OFT [Two-tailed unpaired *t*-test]. **(H)** Representative traces during EPMT between the two groups. **(I-K)** Entry times **(I)** and duration **(J)** in the open arms, as well as anxiety index **(K)** of two groups in the EPMT [Two-tailed unpaired *t*-test]. **(L)** Representative traces during TCT between the two groups. **(M, N)** Sniffing time **(M)**, and social index **(N)** of two groups in the TCT [**(M)** Two-tailed unpaired *t*-test: mCherry: E *vs* S1, *t*_(18)_ = 6.5400, *p* < 0.0001; hM3Dq: E *vs* S1, *t*_(24)_ = 7.5000, *p* < 0.0001]. mCherry: n = 10 mice, hM3Dq: n = 13. Data represent the *mean* *±* *SEM*. ^*^p < 0.05, ^**^p < 0.01, ^***^p < 0.001, ^****^p < 0.0001.

If taVNS is sufficient to saturate synaptic functions of the glutamatergic TANs^ACC^ after exposure to mSPS, as confirmed by previous results of mEPSC and pre-LTP, there would be ceiling effects of taVNS on improving the anxiety-like behaviors in PTSD-like mice. To verify this conjecture, we employed a Gq-coupled chemogenetic strategy to activate the glutamatergic TANs^ACC^ in Fos-TRAP2 mice (Fig. 6A) and then observed anxiety-like behaviors in mSPS+taVNS mice. As shown in Fig. 6D–N, no significant differences were observed in hM3Dq mice in their performance of OFT, EPMT, and TCT when compared to the mCherry group subjected to mSPS exposure and taVNS interventions. Combined with the results obtained in Fig. 4, these data provide strong evidence that the presynaptic glutamate transmission of the glutamatergic TANs^ACC^ in mSPS+taVNS mice might have been saturated after receiving taVNS treatments. Therefore, artificially reactivating these neurons is no longer able to further heighten the effects of taVNS.

## Discussion

PTSD is a disorder caused by exposure to a series of traumatic events, which involves a pathological response with behavioral, emotional, functional, and physiological components [33, 34]. Traumatic events may cause post-traumatic distress and anxiety, with anxiety ultimately exacerbating negative reactions [2]. Previous clinical studies have described the phenomenon of anxiety among patients with PTSD, which suggests that interventions to diminish anxiety might be effective in decreasing PTSD symptoms [2, 35]. VNS is a type of physical therapy that can be used in the treatment of both psychiatric and neurological disorders [36]. Although the positive effects of VNS on PTSD patients and animal models have received research attention, the role of AVN stimulation in PTSD-related anxiety has been overlooked, and its precise cell-type organization and activity-dependent functions have remained unknown.

AVN stimulation has been used as a healing practice for over 2500 years [37, 38]. Therefore, the proposal for applying taVNS may be considered novel; nevertheless, its use and development are quite ancient [39]. In this paper, our study demonstrates that taVNS improved anxiety-like behaviors and social interaction deficits in PTSD-like mice compared to sham stimulation conditions. Consistent with previous studies about AVN stimulation to improve emotional state [37], these results indicate that taVNS has direct positive effects on PTSD-like mice. In addition, taVNS was performed under a low dose of isoflurane to prevent alterations that activated the AVN pathway of animal models. This anesthesia condition did not cause any changes in PTSD-related anxiety-like behaviors when compared to non-anesthetized mice, as observed in many labs’ research [40, 41]. Therefore, this manipulation as a potential bias in our behavior results could be ruled out. Additionally, in this study, all data were collected from male subjects. Given the complex role of gender in PTSD, further characterizing the effects of taVNS between different genders in both animal models and patients is necessary, focusing on aspects such as model paradigms, hormone levels, and shared or sex-specific genotypes.

The AVN primarily consists of afferent fibers, connecting it to the main branch of the VN [40]. Like the VN, the AVN comprises myelinated A-fibers and unmyelinated C fibers [42]. Thus, stimulating the AVN can activate the myelinated A-fibers, which carry the afferent signal from the peripheral nerves to the NTS, modulating the activity of ACC [4, 13]. The ACC, which is a part of the ‘vagal-cortical pathways’, plays a crucial role in emotional and behavioral modulation [43]. Several studies have observed the decreased gray matter volume and inactivation of ACC in individuals with PTSD [44–46], which is an acquired feature of this disease. Activation of the ACC is inversely associated with the severity of PTSD symptoms [43, 44]. A neuroimaging study has demonstrated that stimulating specific regions of the ear thought to have AVN innervations can activate afferent vagal networks and increase the bilateral activation of the ACC [47]. The cFos protein is an indicator of cell activation in response to external stimuli. Consistent with these studies, our results confirm that stimulating the AVN with the taVNS device can activate specific groups of neurons in the ACC of PTSD-like mice, which is identified through the Fos-TRAP labeling method. If we selectively ablate these TANs^ACC^, the positive effects of taVNS on anxiety-like behaviors and social interaction deficits will be abolished (Fig. S6). Therefore, these findings imply a necessary relationship between the enhancement of the functional activity of TANs^ACC^ and the positive effects of taVNS on PTSD-related behaviors.

ACC is a heterogeneous structure constituted of two types of neurons: glutamatergic neurons, also known as pyramidal neurons, as well as GABAergic interneurons, which are classified into somatostatin, vasoactive intestinal polypeptide, and parvalbumin interneurons [48]. The distinct alterations of Glu^ACC^ and GABA^ACC^ neurons in response to PTSD symptoms have been reported by many laboratories. Individuals with PTSD have been found to exhibit increased levels of GABA transmission in the ACC [49]. Similarly, PTSD-like mice display overactivation of PV interneurons in the ACC, which is responsible for delayed fear memory extinction [50, 51]. Other studies have reported a lower number of neuronal dendritic branches and spines, as well as decreased glutamate receptor expressions in the ACC of rats with PTSD [43]. However, most reports focus exclusively on the role of Glu^ACC^ or GABA^ACC^ neurons in the development of PTSD and haven’t explicitly addressed the cellular composition of ACC populations associated with AVN stimulation in PTSD individuals and animals, which might be crucial in correcting PTSD-related anxiety behavior. To determine the dominant neurons concerned with the positive effects of taVNS on PTSD-like mice, we used immunofluorescent staining co-localized with TANs^ACC^. Our findings show that taVNS-recruited glutamatergic neurons in ACC are significantly greater than those of GABAergic neurons in PTSD-like mice. If we specifically inhibit these glutamatergic TANs^ACC^, then the positive effects of taVNS on anxiety-like behaviors will be diminished in PTSD-like mice. However, the effect of taVNS on social interaction deficits is not affected. These findings are not entirely consistent with the results obtained from the basal behavioral assessments (Fig. 1 and Fig. S6), which may be because inhibiting these tagged neurons only causes the suppression of glutamatergic TANs^ACC^, the partially labeled GABA^ACC^ neurons remain functional. GABAergic neurons in the ACC are important in modulating social interaction behaviors. In particular, parvalbumin interneurons are more responsible for initiating sociability, whereas somatostatin interneurons have a prominent role in social preference [48, 52]. Therefore, when we specifically inhibited the majority of glutamatergic TANs^ACC^ by DREADD, the tagged GABA^ACC^ neurons remained functional. That may be the reason why the social behaviors of mSPS+taVNS mice were not affected, but the positive effects on anxiety-like behaviors were reversed. Future experiments will further elucidate the functional activity of taVNS-activated GABAergic neurons in ACC and confirm its mechanism on PTSD-related social interaction deficits.

The changes in synapse efficacy within diverse brain regions and neural circuits may mediate behavior and cognitive performance, as described by Donald Hebb [53, 54]. When experiencing traumatic stress, subjects with PTSD have a damaged glutamatergic system, which thereby leads to inadequate glutamate release, implying an excitatory neurotransmission deficit [43]. Previous studies have demonstrated that AVN stimulation can promote neural plasticity by releasing neuromodulators in the brain, enhancing LTP, and increasing the expression of neurotrophic factors, such as BDNF [55]. Moreover, stimulation of the AVN significantly alters calcium signaling, which plays a pivotal role in regulating depolarization signaling and the activity of synapses in the ACC region [8]. Consistent with these findings, we found that treatment with taVNS is sufficient to activate ACC neurons, which could be further reactivated during the anxiety-related behavioral tests. Moreover, we compared the basal synaptic transmission of tagged and non-tagged neurons in ACC and observed that tagged neurons show a significantly increased mEPSC frequency and decreased PPR relative to non-tagged neurons. These results indicate that the increased presynaptic probability of glutamate release in the glutamatergic TANs^ACC^ is responsible for the potentiation of basal synaptic transmission in mSPS+taVNS mice, which may explain the positive effects of taVNS on PTSD-related anxiety-like behaviors.

There are two main forms of LTP in ACC synapses, including NMDA receptor (NMDAR)-dependent postsynaptic long-term potentiation (post-LTP) and NMDAR-independent presynaptic LTP (pre-LTP) [9, 56]. Post-LTP is associated with chronic pain [57], whereas pre-LTP is considered to be correlated with anxiety-like behavior (Fig. S7) [32, 58]. There is a series of studies that have elaborated the mechanisms of depotentiation from potentiated synaptic strength, involved in physiological and pathological states [18]. For instance, when the synaptic transmission levels of glutamate in the hippocampus have been improved to a potentiated level, subsequent suffering events or stimulation protocols that typically make for post-LTP can only induce further depotentiation [59, 60]. Moreover, except for post-LTP, if the expression of the LTP component is presynaptic, then it is also possible for that presynaptic component to undergo depotentiation (Fig. S8) [61, 62]. In this paper, we hypothesize that taVNS acts as a priming signal that contributes to potentiated neuronal activity, leaving a long-lasting trace that influences the subsequent plasticity induction. To identify this hypothesis, we conducted a well-described in vitro protocol, which has been reported to be applied in the hippocampus and amygdala to induce pre-LTP, in the glutamatergic TANs^ACC^, and the DREADD-activation in vivo strategy to regulate the behaviors of mSPS+taVNS mice. The results indicate that the enhancement of the presynaptic release probability induced by taVNS occludes or inhibits further synaptic potentiation produced either by low-frequency stimulation or DREADD-activation of the glutamatergic TANs^ACC^.

Together, these results accurately anatomize the functional relationship between the positive effects of taVNS on PTSD-related anxiety behaviors and the activity of glutamatergic TANs^ACC^. Nevertheless, several crucial questions warrant further elucidation in subsequent studies, such as the origins of the presynaptic projection of the glutamatergic neurons in ACC, whether these sites are also the regulatory targets of taVNS, and the specific molecular mechanism underlying presynaptic potentiation.

### Limitations

The application of the taVNS device to mice in this work was conducted under anesthesia within our laboratory experimental setting, unlike an implantable or external VNS used in other rodent studies performed under non-anesthetized conditions. Furthermore, we only investigated male mice to evaluate the potential positive effects of taVNS on anxiety-like behaviors in a PTSD-like model.

## Supplementary information

Supplemental Material

## Data Availability

The original contributions presented in the study are included in the article. Further reasonable inquiries can be directed to the corresponding author.
