# Supplementary material for: Transcutaneous auricular vagus nerve stimulation alleviates anxiety-like behaviors in mice with post-traumatic stress disorder by regulating glutamatergic neurons in the anterior cingulate cortex
Source: Transl Psychiatry. 2025 Aug 23;15:313. doi: 10.1038/s41398-025-03535-9 (PMC12375034; doi:10.1038/s41398-025-03535-9)
Supplement: Supplementary file 1 — Supplemental Material [file 41398_2025_3535_MOESM1_ESM.pdf]

Supplementary Information:

Transcutaneous auricular vagus nerve stimulation alleviates anxiety-like behaviors in mice with post-traumatic stress disorder by regulating glutamatergic neurons in the anterior cingulate cortex

Zhijun Diao [1, 2, 3], Yan Zuo [4], Jinming Zhang [5], Ke Chen [4], Yongbin Liu [4], Yuwei Wu [1, 2, 3], Feng Miao [6], Haifa Qiao [1, 2, 3, ✉]

[1] Institute for Chinese Medicine Frontier Interdisciplinary Science and Technology, Shaanxi University of Chinese Medicine, Xianyang, Shaanxi Province, China, 712046.

[2] Shaanxi Key Laboratory of Integrative Acupuncture & Medicine, Shaanxi University of Chinese Medicine, Xianyang, Shaanxi Province, China, 712046.

[3] Key Laboratory of Acupuncture and Neurobiology, Shaanxi Administration of Traditional Chinese Medicine, Xianyang, Shaanxi Province, China, 712046.

[4] College of Acupuncture-moxibustion and Tuina, Shaanxi University of Chinese Medicine, Xianyang, Shaanxi Province, China, 712046.

[5] Key Laboratory of Modern Teaching Technology, Ministry of Education, Shaanxi Normal University. Xi'an, Shaanxi Province, China. 710062.

[6] The Second Affiliated Hospital of Shaanxi University of Chinese Medicine, Xianyang, Shaanxi Province, China, 712020.

✉ Corresponding author:

Haifa Qiao, PhD. Email: qiaohaifa@sntcm.edu.cn. Tel: +86(029)38185032. Fax: +86(029)38185333. Address: 1 Century Ave., Qindu District, Xianyang, Shaanxi Province, China. Code: 712046.

Materials and Methods

Animal model induced by mSPS

In brief, the procedure commenced with the confinement of mice for 4 h, followed by a 15-minute rest period within their home cages. Then, these mice were compelled to swim for 20 min in a plastic tube filled to approximately two-thirds capacity with water. Subsequently, the mice were anesthetized with ether until they became unconscious, as evidenced by accelerated respiration and a lack of responses to toe and tail pinches. Following a 15-minute recuperation period, the mice endured a single unconditioned foot shock (0.8 mA, 5 s) in the chamber equipped with an electrical grid floor. Then, the mice returned to their home cages and remained undisturbed for 7 days. For the control procedure, mice were left in their home cages devoid of water and food while the model mice underwent mSPS.

4-OHT preparation

Briefly, 4-OHT was solubilized in ethanol at a concentration of 20 mg/ml by agitating the solution at 37° C for 15 min, followed by aliquoting and storing at -20° C for several weeks. Before use, the 4-OHT was redissolved in ethanol and agitated again at 37° C for 15 min. Subsequently, corn oil (Sigma, Cat#s259853) was incorporated to achieve a final concentration of 10 mg/ml 4-OHT, evaporating the ethanol. The resultant 4-OHT solutions were stored at 4° C and used within 24 h.

Behavioral Tests

Mechanical and Thermal Sensitivity

The experiment was performed as previously described with modifications [1-2]. After 1 day of acclimatization to the experimental environments, the four

groups of mice were assessed for mechanical withdrawal threshold and thermal withdrawal threshold latency. Mechanical sensitivity was examined utilizing the Electronic von Frey Anesthesiometer (IITC, USA) filament, with mice placed individually into a transparent plastic chamber with a wire mesh grid to allow for calibrated Von Frey hairs insertion on the surface of the hind paw. The mechanical withdrawal threshold (in grams) was recorded when the mouse withdrew its hind leg in response to von Frey stimulation. Thermal withdrawal latency was measured by heating the hind paws with the Hargreaves radiant heat apparatus (IITC, USA) and was taken down when the mouse exhibited hind paw withdrawal. The mean threshold was calculated from five applications.

Gait analysis

The experiment was conducted as previously described with modifications [3-5]. Gait was recorded during a mouse's spontaneous walk using an automated Noldus CatWalk XT system (Noldus, Netherlands). The apparatus comprises a 1.5-meter-long glass corridor illuminated by subdued green light projected onto the glass walkway and placed in a dark and silent room. Employing Illuminated footprints technology, paw movements were captured by a high-speed video camera positioned underneath the glass. Each mouse was assessed individually over three consecutive runs. Mice exhibiting hesitation during the beam walk or attempting to reverse direction were excluded from the final analysis. All gait parameters were analyzed with a special focus on (1) Sequence regularity index (SRI): a percentage index measuring interlimb coordination, calculated based on the number of normal step sequence patterns (NSSP), number of paws, and paw placements, SRI = 100% × (NSSP × no. of paws) / no. of paw placements; (2) Brake duration: Time of the braking

portion of the stance phase; (3) Propulsion duration: Time of the propelling portion of the stance phase; (4) Mean intensity: Average pressure exerted by a single paw on floor contact; (5) Swing time: Time of paw spent in the air between two consecutive steps; (6) Swing speed: parameter calculated using swing and stride length; (7) Stride length: distance between paw placement in two consecutive steps of the same paw; (8) Body speed: walk speed measured as distance over time; (9) Print length: measured length of the print area; (10) Print width: measured width of the print area.

Immunofluorescence

Mice were deeply anesthetized using pentobarbital sodium (i.p., 20 mg/kg) and then perfused with saline after behavioral assessments. Their brains were extracted and postfixed in 4% paraformaldehyde at 4°C overnight before being subjected to gradient dehydration in 20% and 30% sucrose solutions until they sank to the bottom of the container. Coronal slices (25 μm for cFos staining, 40 μm for other experiments) were prepared using a cryostat microtome (HM525 NX UV, Leica, Germany). After washing with phosphate-buffered saline (PBS), sections encompassing the ACC region were incubated with 0.5% Triton X-100 and 10% normal donkey serum for 2 h. Then, primary antibodies, including anti-NeuN (1:500, rabbit, mAb#24307, Cell Signaling Technology), anti-glutamate (1:250, rabbit, Cat#G6642, Sigma), anti-GABA (1:250, rabbit, Cat#A2052, Sigma), and anti-c-Fos (1:500, rabbit, mAb#2250, Cell Signaling Technology), mixed with 0.3% Triton X-100 and 5% normal donkey serum, were applied at 4°C for overnight. Sections were then treated with the Alexa Fluor 488 conjugated anti-rabbit secondary antibody (1:500, Cat#711-545-152, Jackson ImmunoResearch) for 2 h. Subsequently, slides were mounted with antifade

reagents. Fluorescence signals were observed using a Leica TCS SP8 confocal microscope or a SLIDEVIEW™ VS200 research slide scanner. Image analysis was performed using Image J software (NIH, Bethesda, MD, USA) with manual quantification from three to five sections per mouse. All quantitative analyses were performed blindly.

Electrophysiological recording

Mice were anesthetized with urethane and decapitated. Coronal slices, 300 μm in thickness, were precisely sectioned using a VT1200S microslicer (Leica, Germany), and then collected in a holding chamber containing oxygenated artificial cerebrospinal fluid (ACSF) with the following composition (in mM): 125 NaCl, 2.5 KCl, 25 glucose, 25 $NaHCO_3$, 1.25 NaH_2PO_4, 2 $CaCl_2$, and 1 $MgCl_2$, gassed with 5% CO_2 /95% O_2 at 32-34° C for at least 1 h before use. Each slice was transferred to a recording chamber and continuously perfused with oxygenated ACSF throughout the experiment. During voltage-clamp recordings, pipettes had 3-5 MΩ resistance when filled with an intracellular solution composed of (in mM): 140 $CsCH_3SO_3$, 10 HEPES, 2 QX-314, 2 $MgCl_2$, 0.2 EGTA, 4 MgATP, 0.3 Na_2GTP, 10 Na_2-phosphocreatine (pH 7.2-7.4 with CsOH, osmolality between 285-290 mOsm). The recording commenced 5 min post membrane disruption to ensure equilibrium between the internal solution and the intracellular fluid. Whole-cell recordings of ACC neurons were conducted using a Multiclamp 700B amplifier and a Digidata 1550B (Molecular Devices, USA), with data acquisition and analysis facilitated by pClamp 10.5 software (Molecular Devices, USA). All experiments were carried out with 100 μM picrotoxin (PTX, MCE, USA). Series and input resistances were consistently monitored, discarding any data exhibiting

resistance fluctuations exceeding 20%. Data were filtered at 1 kHz and digitized at 10 kHz. All electrophysiological data analyses were conducted blinded to the identity of experimental groups.

To ascertain the efficacy of DREADD expression, changes in membrane potential to the designer receptor agonist CNO under the current-clamp mode (I=0 pA) were assessed. The pipettes had 4-6 MΩ resistance after filling with potassium-based intracellular solution, comprising (in mM) 130 K-gluconate, 5 KCl, 2 $MgCl_2$, 10 HEPES, 0.6 EGTA, 0.3 Na-GTP, and 2 Mg-ATP (pH 7.2-7.4 with KOH, osmolality of 285-290 mOsm).

Supplementary References

1. Zhu X, Zhou WJ, Jin Y, Tang HD, Cao P, Mao Y, et al. A central amygdala input to the parafascicular nucleus controls comorbid pain in depression. Cell Rep. 2019; 29: 3847-3858.

2. Diao ZJ, Di YY, Wu ML, Zhai C, Kang M, Li Y, et al. Single Exposure to Cocaine Impairs Reinforcement Learning by Potentiating the Activity of Neurons in the Direct Striatal Pathway in Mice. Neurosci Bull. 2021; 37: 1119-1134.

3. Pitzer C, Kurpiers B, Eltokhi A. Gait performance of adolescent mice assessed by the CatWalk XT depends on age, strain, and sex and correlates with speed and body Weight. Sci Rep. 2021; 11: 21372.

4. Matas E, Maisterrena A, Thabault M, Balado E, Francheteau M, Balbous A, et al. Major motor and gait deficits with sexual dimorphism in a Shank3 mutant mouse model. Mol Autism. 2021;12: 1-16.

5. Garrick JM, Costa LG, Cole TB, Marsillach J. Evaluating gait and locomotion in rodents with CatWalk. Current Protocols. 2021; 1: e220

Supplementary Figures

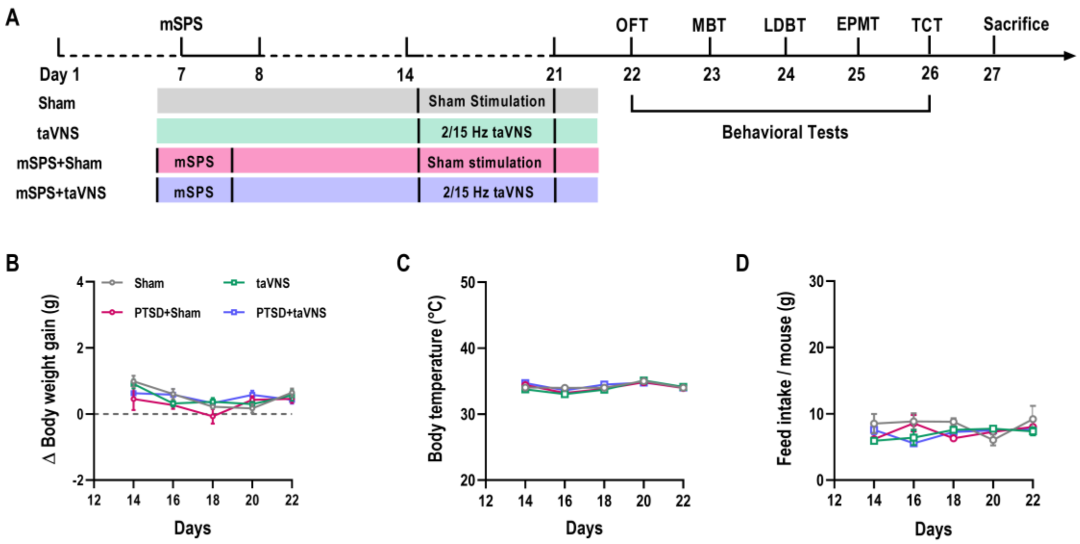

Fig. S1 The well-being assessments during the taVNS period across the four groups of mice. (A) Experimental process diagram. **(B-D)** Body weight gain **(B)**, body temperature **(C)**, and feed intake **(D)** of each mouse during the taVNS intervention period. n = 10 mice per group. Data represent the *mean* ± *SEM*.

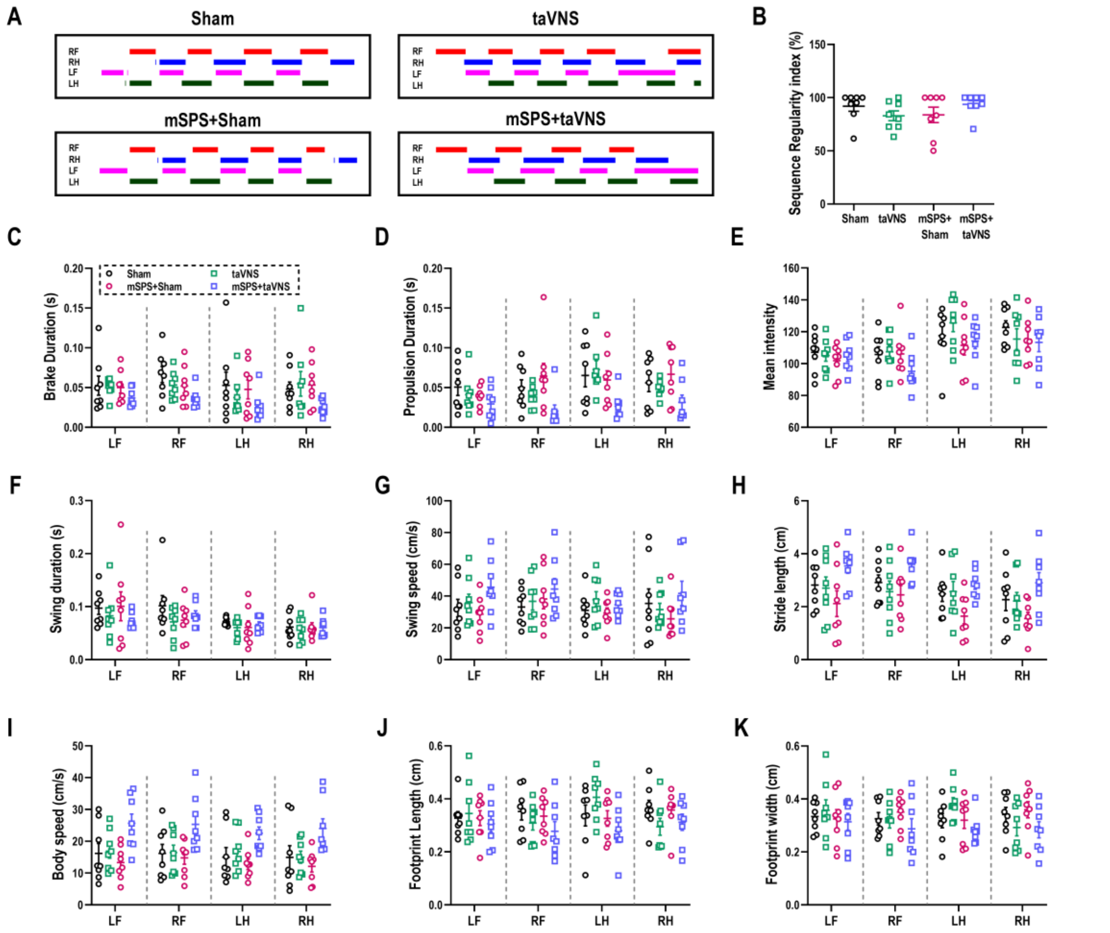

Fig. S2 Exposure to mSPS or taVNS treatment did not change the gait in mice. (A) Representative timing view of footprints. **(B)** Sequence regularity index. **(C)** Brake duration. **(D)** Propulsion duration. **(E)** Mean intensity. **(F)** Swing duration. **(G)** Swing speed. **(H)** Stride length. **(I)** Body speed. **(J)** Footprint length. **(K)** Footprint width. n = 8 mice per group. Data represent the *mean* ± *SEM*.

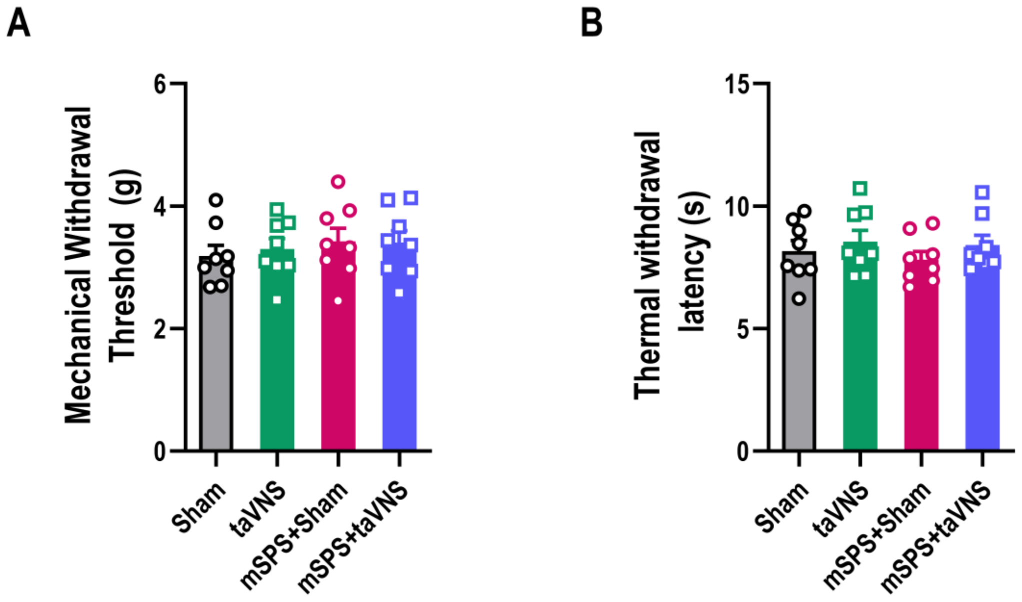

Fig. S3 The effects of taVNS on anxiety-like behaviors in PTSD-like mice were not due to its impact on pain responses. (A) The mechanical withdrawal threshold. **(B)** The thermal withdrawal latency. n = 8 mice per group. Data represent *mean* ± *SEM*.

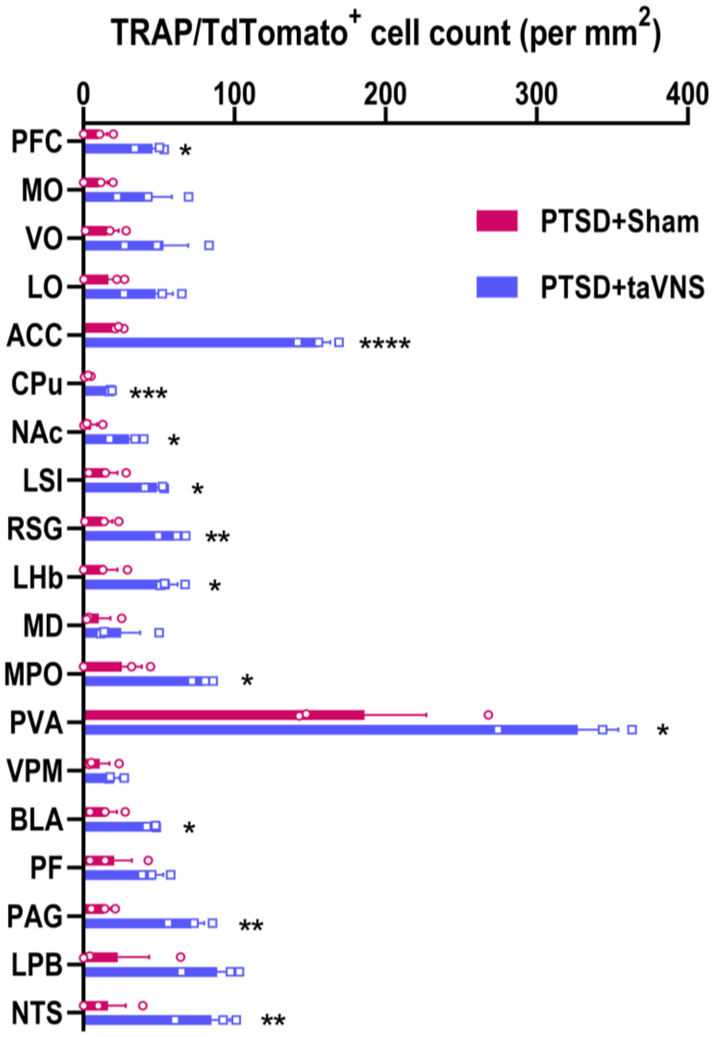

Fig. S4 Differences in TRAP/TdTomato^{+} cell counts across brain regions in PTSD+sham *versus* PTSD+taVNS groups. [Two-tailed unpaired *t*-test. PFC: $t_{(4)} = 4.0470$, $p = 0.0155$; MO: $t_{(4)} = 2.3360$, $p = 0.0797$; VO: $t_{(4)} = 2.0670$, $p = 0.1077$; LO: $t_{(4)} = 2.249$, $p = 0.0877$; ACC: $t_{(4)} = 16.3600$, $p < 0.0001$; CPu: $t_{(4)} = 11.85$, $p = 0.0003$; NAc: $t_{(4)} = 3.246$, $p = 0.0315$; LSI: $t_{(4)} = 4.0240$, $p = 0.0158$; RSG: $t_{(4)} = 5.5680$, $p = 0.0051$; LHb: $t_{(4)} = 4.402$, $p = 0.0117$; MD: $t_{(4)} = 1.005$, $p = 0.3716$; MPO: $t_{(4)} = 3.6560$, $p = 0.0217$; PVA: $t_{(4)} = 2.8760$, $p = 0.0452$; VPM: $t_{(4)} = 1.3260$, $p = 0.2554$; BLA: $t_{(4)} = 4.3300$, $p = 0.0123$; PF: $t_{(4)} = 2.0930$, $p = 0.1045$; PAG: $t_{(4)} = 6.0080$, $p = 0.0039$; LPB: $t_{(4)} = 2.7440$, $p = 0.0517$; NTS: $t_{(4)} = 4.6530$, $p = 0.0096$]. n = 3 mice per group. Data represent *mean ± SEM*. $^{*}p<0.05$, $^{**}p<0.01$, $^{***}p<0.001$, $^{****}p<0.0001$.

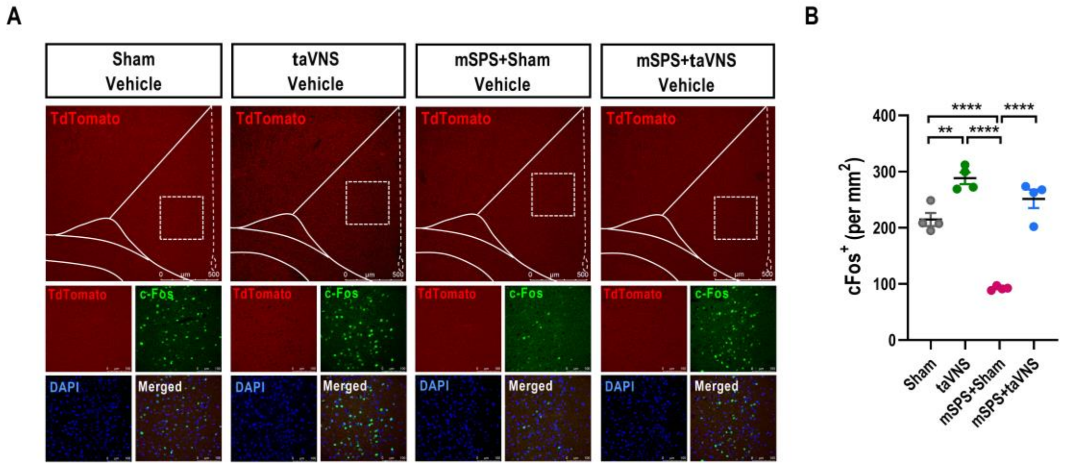

Fig. S5 The involvement of ACC in the EPMT among the Sham, taVNS, mSPS+Sham, and mSPS+taVNS groups with vehicle injections. (A) Top: Representative images of the ACC region from RosaFos2 mice with vehicle injections among the Sham, taVNS, mSPS+Sham, and mSPS+taVNS groups. Scale bar, 500 μm. Bottom: cFos induction following the EPMT in the ACC. Scale bar, 100 μm. **(B)** The number of $cFos^+$ cells [Two-way RM ANOVA with *Tukey*'s multiple comparisons tests, n = 4 mice per group: $F_{(1, 12)} = 13.99$, $p = 0.0028$; Sham Vehicle vs taVNS Vehicle, $p = 0.0033$; Sham Vehicle *vs* mSPS+Sham Vehicle, $p < 0.0001$; taVNS Vehicle *vs* mSPS+Sham Vehicle, $p < 0.0001$; mSPS+Sham Vehicle *vs* mSPS+taVNS Vehicle, $p < 0.0001$]. Data represent the *mean* ± *SEM*. $^*p<0.05$, $^{**}p<0.01$, $^{***}p<0.001$, $^{****}p<0.0001$.

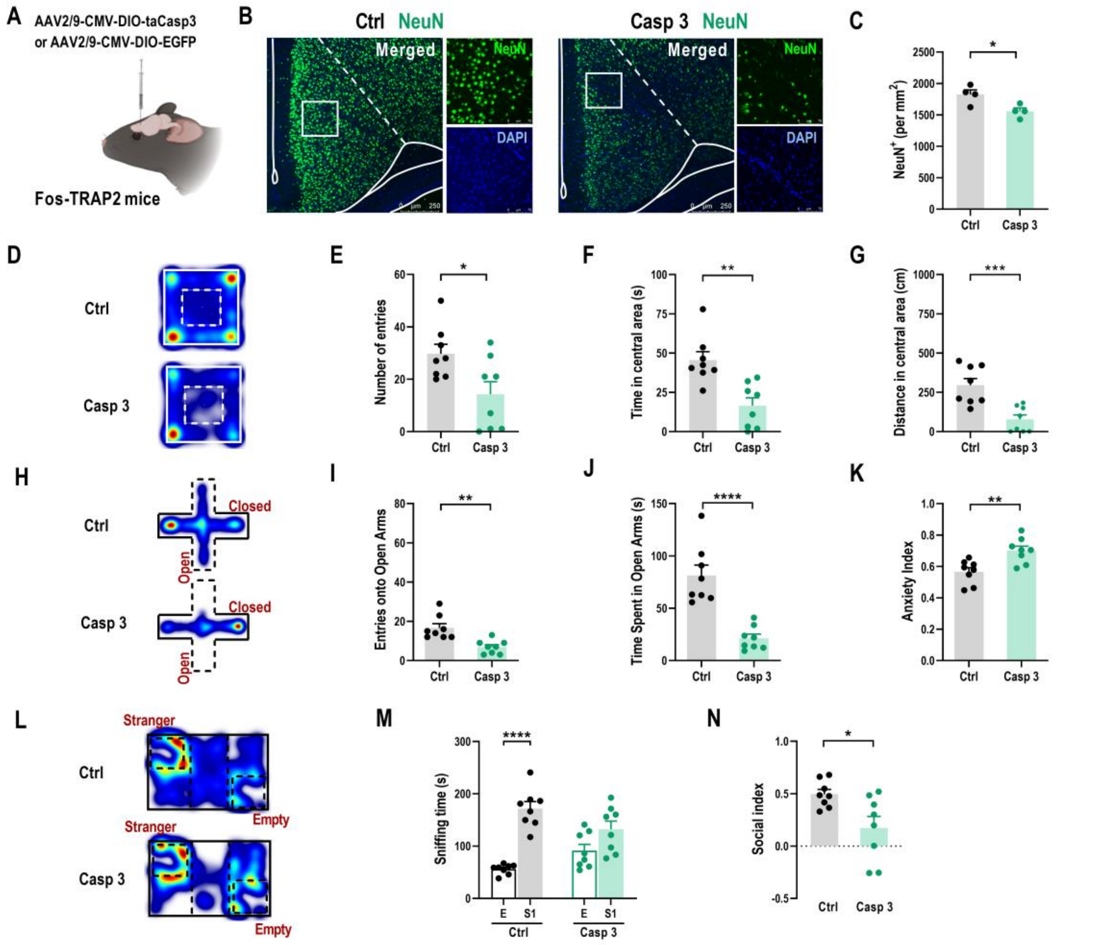

Fig. S6 Selective induction of TANs^{ACC} apoptosis of PTSD-like mice suppressed the effects of taVNS on PTSD-related anxiety-like behaviors and social interaction deficits. (A) Schematic of AAV-CMV-DIO-taCasp3 or AAV-CMV-DIO-EGFP injection into the ACC of Fos-TRAP2 mice (Created with BioRender.com, 2025). **(B)** Representative images of NeuN immunofluorescence in the Fos-TRAP2 mice with or without taCasp3 virus, scale bar, 250 μm (left) and 75 μm (right). **(C)** The number of $NeuN^+$ cells [n = 4 mice per group. Two-tailed unpaired *t*-test: $t_{(6)} = 2.930$, $p = 0.0263$]. **(D)** Representative traces during OFT in Ctrl and Casp 3 mice. **(E-G)** Entry times **(E)**, duration **(F)**, and traveled distance **(G)** in the central area of the open field of two groups [n = 8 mice per group. Two-tailed unpaired *t*-test: **(E)** $t_{(14)} = 2.580$,

p = 0.0218. **(F)** $t_{(14)}$ = 3.941, p = 0.0015. Mann-Whitney U test: **(G)** Z = -3.0456, p = 0.0011]. **(H)** Representative traces during EPMT between the two groups. **(I-K)** Entry times **(I)**, and duration **(J)** in the open arms, as well as anxiety index **(K)** of two groups [n = 8 mice per group. Mann-Whitney U test: **(I)** Z = -3.0614, p = 0.0006. Two-tailed unpaired *t*-test: **(J)** $t_{(14)}$ = 5.586, p < 0.0001. **(K)** $t_{(14)}$ = 3.485, p = 0.0036]. **(L)** Representative traces of two groups during TCT. **(M-N)** Sniffing time **(M)**, and social index **(N)** of two groups [n = 8 mice per group. **(M)** Ctrl: E *vs* S1, Mann-Whitney U test, Z = -3.3607, p = 0.0002; Casp 3: E *vs* S1, Two-tailed unpaired *t*-test, $t_{(14)}$ =2.0980, p = 0.0545. **(N)** Mann-Whitney U test: Z = -2.2054, p = 0.0281]. Data represent the *mean* ± *SEM*. $^{*}p$<0.05, $^{**}p$<0.01, $^{***}p$<0.001, $^{****}p$<0.0001.

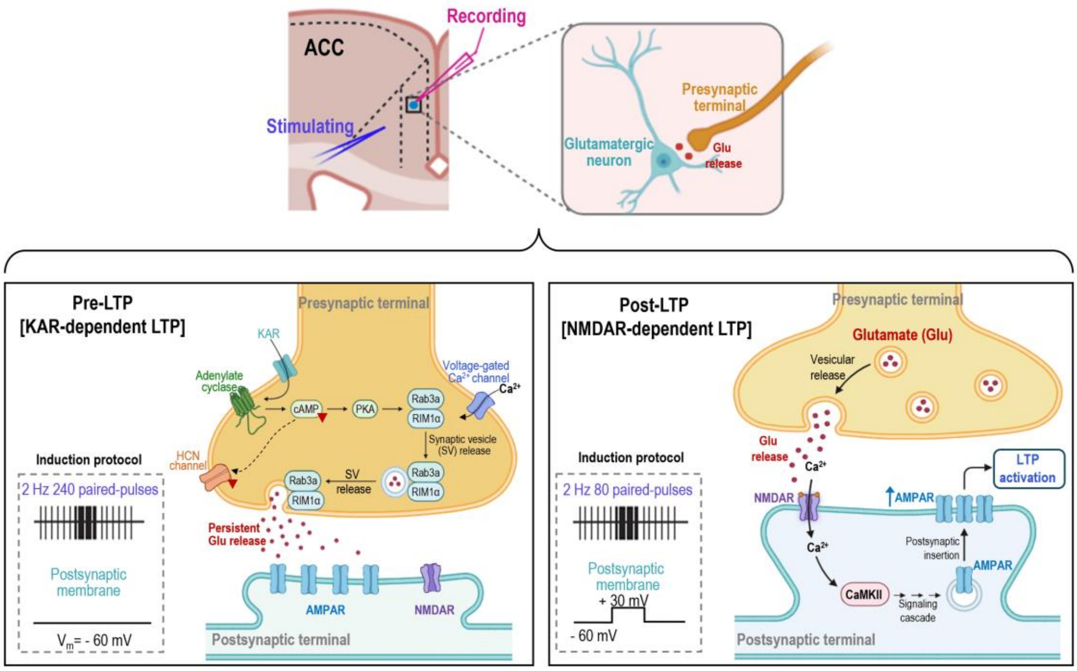

Fig. S7 The schematic diagram explaining Pre-LTP and Post-LTP mechanisms. In the ACC, there are two main forms of LTP, including NMDA receptor (NMDAR)-dependent postsynaptic long-term potentiation (Post-LTP) and NMDAR-independent presynaptic LTP (Pre-LTP), also known as kainate receptor (KAR)-dependent pre-LTP. Specifically, pre-LTP arises from enhanced presynaptic neurotransmitter release via the KAR/AC1-ERK pathway, whereas post-LTP involves postsynaptic receptor upregulation and structural modifications mediated by NMDAR-cAMP signaling. Both forms of LTP contribute to synaptic plasticity. Moreover, the induction protocols of Pre-LTP and Post-LTP are also distinct: Pre-LTP is induced by 240 paired presynaptic pulses at a 2 Hz frequency under a holding potential of -60 mV, while Post-LTP is initiated by 80 pulses at the same frequency, coupled with postsynaptic depolarization at +30 mV. (Created with BioRender.com, 2025)

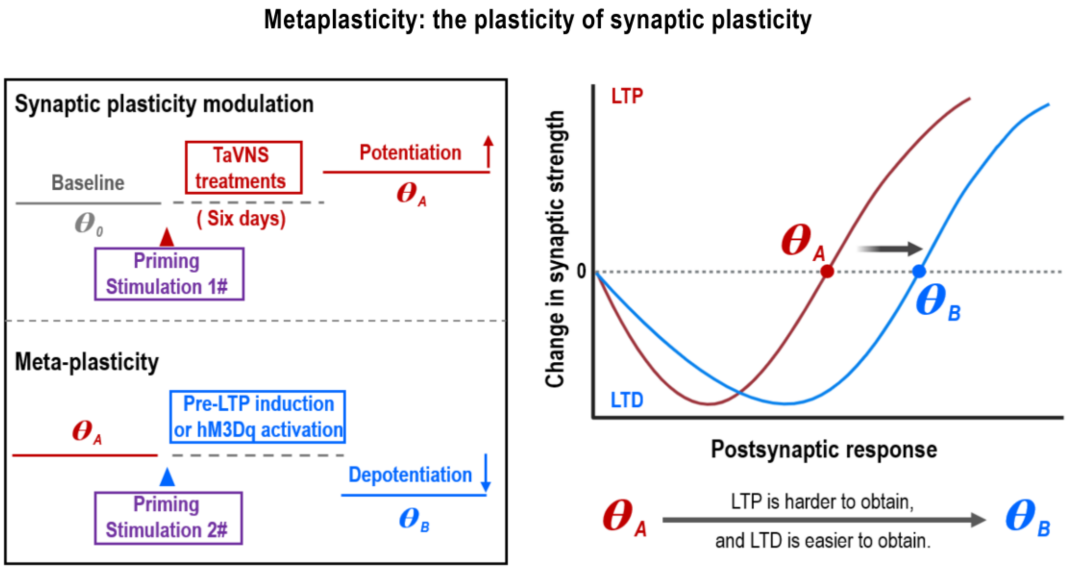

Fig. S8 The mechanism of de-potentiation. Meta-plasticity, a term coined by Abraham and Bear in 1996, refers to the ability of synapses to dynamically adjust their capacity for subsequent LTP or LTD induction based on their recent history of neural activity. It can also be understood as experience-dependent plasticity. For instance, if a priming stimulus, such as taVNS treatment, at the first time-point potentiated the synaptic plasticity, then we can establish a threshold under this condition as θ_A. Next, if we apply this synapse a higher stimulus at this elevated level, such as the 2 Hz low-frequency stimulation protocol or activating Gq-coupled hM3Dq receptor on these ACC neurons, then the previous threshold θ_A will shift to the right, becoming θ_B, making LTP harder to achieve and LTD more accessible, which is termed de-potentiation. (Created with BioRender.com, 2025)
